# Major Antioxidants and Methods for Studying Their Total Activity in Milk: A Review

**DOI:** 10.3390/mps8060139

**Published:** 2025-11-10

**Authors:** Sergei Yu. Zaitsev

**Affiliations:** Federal Research Center for Animal Husbandry Named After Academy Member L.K. Ernst, Dubrovitsy 60, 142132 Podolsk, Moscow Region, Russia; s.y.zaitsev@mail.ru; Tel.: +7-49567-651363

**Keywords:** cow’s milk, antioxidant activity, analysis and methods, redox titration, total amount of water-soluble antioxidants

## Abstract

The presence of antioxidants in food contributes to the preservation of its taste and technological qualities, preventing its spoilage for a longer time, which is important at all stages of production and storage. The major antioxidants are vitamins, proteins (primarily, enzymes), peptides, amino acids, fatty acid residues of lipids, etc. There is currently an explosive growth in the development of methods for assessing the content and effectiveness of particular antioxidants but not the total antioxidant activity (AOA) in raw milk and food systems. This article provides a critical overview of the most important AOA methods, their mechanisms and applicability, advantages, and limitations (primarily, for antioxidants of milk and dairy products). Among all the antioxidant indicators of milk, the simplest and sufficiently informative is the detection of the total amount of water-soluble antioxidant (TAWSA), which is confirmed by comparison of numerous publications and practical results of various methods (as summarized in this review). It is important to emphasize that the TAWSA of milk is an “integral characteristic” of the most valuable biosubstances (possessing AOA) together. Therefore, the TAWSA method is recommended for assessing AOA in raw milk as an “integrated indicator” in dairy husbandry.

## 1. Introduction

Milk is one of the main food products for both humans and many animals, as it contains many important biologically active substances (BASs) [1,2,3,4]. The importance of this product is evidenced by the continuous growth in milk production around the world, that is, from 579.6 million tons in 2000 to 992.7 million tons in 2025 according to the Food and Agriculture Organization of the United Nations (FAO) [5]. For example, around a 3.3% increase in raw milk production is predicted in Russia as of 2025 (i.e., up to 34.1 million tons [5]), compared to 33.0 million tons in 2023 [2].

The exceptional structural and functional organization of animal milk [6,7,8,9] determines the high nutritional value of this product [3]. Among milk BASs, those substances that have “antioxidant activity” (antioxidants) deserve special attention, because they prevent oxidation of “milk biological structures” and/or destroy the resulting “active radicals” [7]. An assessment of the total amount of antioxidants or, in particular, the total amount of water-soluble antioxidants (TAWSA) (as well as the activity of individual antioxidants in milk) should be accompanied by detailed studies of its biochemical composition [2,3,4,7,8,9]. This is important from both fundamental and applied perspectives [9,10,11,12,13,14,15,16,17,18,19,20,21].

The detailed study of antioxidants in milk from farm animals (depending on some physiological and biochemical parameters as well as the correlations between these parameters) is one of the most important areas of modern research both in Russia [2,7,10,11,12,18,19,20] and worldwide [8,9,13,14,15,16,17]. All of the following parameters (i.e., age, terms and periods of cow lactation, general physiological condition, milking time, feeding, etc.) are important for assessing the antioxidant activity (AOA) of raw milk [21,22,23,24,25,26] and its further processing [27,28,29,30]. Most work in these areas is focused on individual antioxidants [18,19,20,21,22,23,24,25,26] and sometimes in connection with the listed parameters, but rarely for assessing an “integral” AOA parameter such as TAWSA [2,7,10,11,12,17,18].

Moreover, there is currently no single standard indicator of antioxidant activity, and there is no generally accepted terminology [1,2,3,7,8,9,14,15,16,17,20,21,22,23,24,25,26]. For example, there are no methods for determining the antioxidant activity in milk that are mentioned in the current Russia Technical Standards “Raw Cow’s Milk. Technical Conditions” [2] in contrast to the many prescribed methods for measuring about 20 different BAS parameters in raw milk collected from the Russian farms [2].

The aim of the work is a comparative assessment of the major biologically active substances with antioxidant activity and methods for determining the total antioxidant activity of milk based on the analysis of our own data alongside published data. To collect data, we used the following information and analytical systems and websites: The Food and Agriculture Organization (FAO) (https://www.fao.org/statistics/data-collection/general/en (accessed on 1 September 2025)); the Ministry of Agriculture of the Russian Federation (including order No. 82,087 of 7 May 2025 http://publication.pravo.gov.ru/document/0001202505070019 (accessed on 3 September 2025)); FGBNU VNIIPlem (https://vniiplem.ru/breeds/mol-krs/ (accessed on 4 September 2025)); the Federal Research Center for Animal Husbandry named after Academy Member L.K. Ernst (https://www.vij.ru/en/institute/contacts-and-requisitions (accessed on 5 September 2025)); and other organizations. We used databases for searching the scientific literature such as Scopus (https://www.scopus.com/home.uri (accessed on 8 September 2025)), the Web of Science Core Collection (https://access.clarivate.com/ (accessed on 9 September 2025)), Web of Knowledge (https://www.webofknowledge.com, https://www.sciencedirect.com (accessed on 10 September 2025)), the Russian Science Citation Index (RSCI, https://www.elibrary.ru/ (accessed on 11 September 2025)), CyberLeninka (https://www.cyberleninka.ru (accessed on 12 September 2025)), AGRIS, and others (https://www.researchgate.net/publication (accessed on 15 September 2025)).

**Figure 1:**
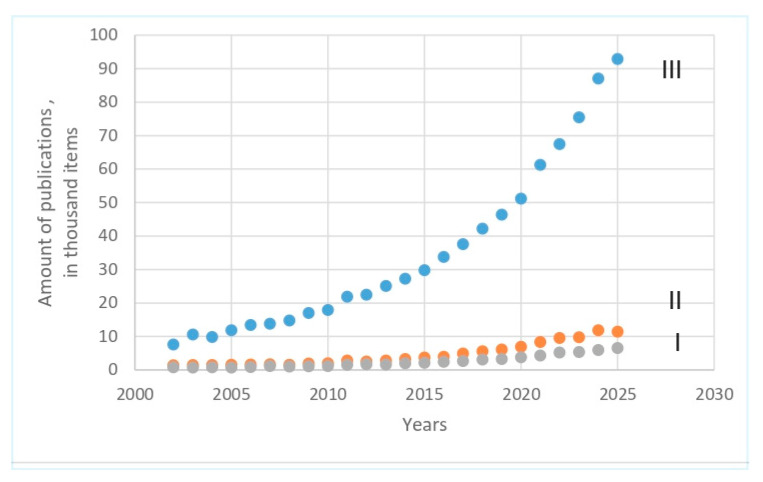
The dynamics of the selected publications found by searching the site “https://www.sciencedirect.com/search system” over the past 20 years (data within 2002–2025): the search was conducted using the following keywords: “Antioxidants in cattle milk” (I, grey dots); “Antioxidants in cow milk” (II, brown dots); “Antioxidants in milk” (III, blue dots).

To gain a comprehensive understanding of the author’s field of interest, it was important to compare the dynamics of publication data in the “https://www.sciencedirect.com/search system” (accessed on 30 September 2025) over the past 20 years. The search was conducted using the following keywords (“Find articles with these terms”): (I) “Antioxidants in cattle milk” (7283 articles in total); (II) “Antioxidants in cow milk” (12,782 articles in total); (III) “Antioxidants in milk” (93,075 articles in total) (accessed on 30 September 2025). This demonstrates not only the enormous number of publications in this field but also the importance of formulating the query as clearly as possible, because a comparison of the numbers for positions I and II clearly shows a difference of 75.5%, which is the price of replacing one word (“cattle” with “cow”). Clearly, the general query using all keywords yielded a huge number of publications, which would have been very difficult to manage within a realistic timeframe. Therefore, the author deliberately limited the search results to about 10% of the major publications of all keyword types for further consideration in this review (Figure 1).

On the other hand, the majority of publications (found by the database check) relate to antioxidant supplementation of animal feed and the evaluation of both individual organ and tissue parameters (primarily blood), as well as animal physiological and biochemical status (PhBS) as a whole. Only a few such publications were reviewed here, particularly those that addressed cow productivity, especially when at least some individual milk parameters were included in the overall data.

## 2. General Approaches to the Classification of Various Antioxidants

In the most general terms, an antioxidant can be any substance that “significantly delays or inhibits oxidation” of biologically active substances of organs and tissues, although it is present there in very low concentrations [7,8,9,14,15,16,17,18,19]. “Reactive oxygen species” (ROS) such as hydroxyl radicals (HO^•−^), superoxide radicals (O_2_^•−^), hydroperoxyl radicals (HOO^•−^), lipid radicals (L^•^), lipid peroxyl radicals (LOO^•^), peroxyl radicals (ROO^•^), lipid alkoxyl radicals (LO), nitrogen dioxide radicals (NO_2_^•^), nitric oxide radicals (NO^•^), thiyl radicals (RS^•^), and even amino acid (AA^•^) radicals coming both from free AA or polypeptide chains (AA^•^) can be found in nature [7,8,9,17,18,19,20,21,22,23,24,25,26,27,28,29,30,31]. There are a fairly large and varied number of antioxidants in milk (Table 1). Vitamins (A, C and E), proteins (including enzymes and some hormones), peptides and amino acids, fatty acid residues of lipids, and a number of low-molecular-mass antioxidants [1,2,3,13,14,15,22,23,24,25,27] are fighting with ROS [7,8,9,17,18,19,20,21,31]. 

**Figure 2:**
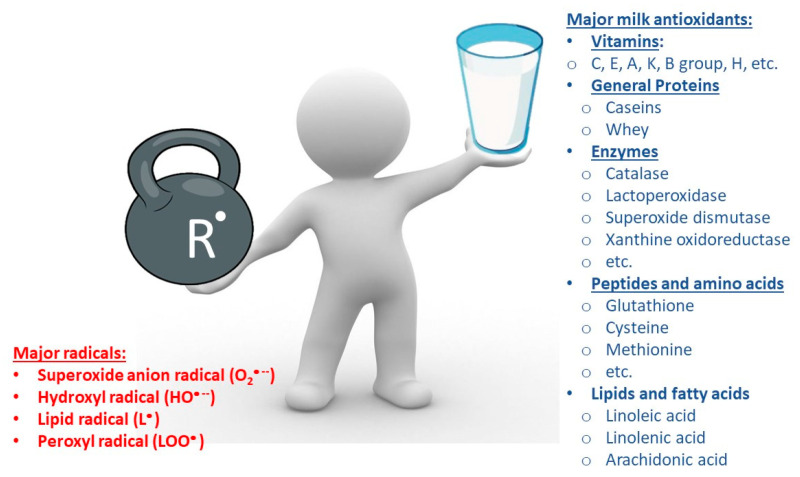
Scheme of the major radicals and antioxidants in milk.

By their chemical nature, antioxidants are a wide class of compounds, including proteins and peptides, phenols and polyphenols (tocopherols, eugenol, pyrocatechin, gallic acid derivatives), flavonoids (rutin, quercetin), steroid hormones, a number of lipids, and many other compounds. Depending on their solubility, fat-soluble (vitamins E, A, K, D) and water-soluble (vitamins C, B_2_, B_6_, B1_2_, peptides, and SH-containing compounds) antioxidants may be distinguished. Distinguished by their molecular weight are of low-molecular-weight (glutathione, ascorbate, β-carotene, α-tocopherol, uric acid) and high-molecular-weight (catalase, peroxidases and others) antioxidants [7,8,9,14,15,16,17,18,19,21,22,23,24,25,26,27,28,29,30]. Another criterion for classifying antioxidants is the place of their formation and their route of penetration into the body. Based on this point, BASs can be classified into exogenous antioxidants (those that enter the body with food) and endogenous antioxidants (those that are synthesized in the body and transported to the site of action by blood) [28,29,30]. Unfortunately, due to the significant diversity of chemical structures and active functional groups of antioxidant molecules, there is still no universal version of their classification, only a general summary (Figure 2).

Even the division of antioxidants into natural and synthetic is quite arbitrary, since many natural BASs are subject to various chemical modifications during the extraction and production of a “pharmacological drug” [30,31,32,33,34]. For example, for pharmacological applications, vitamin E is produced in the form of tocopherol acetate, which does not exhibit antioxidant properties in model experimental systems, but when it enters the body of animals, it quickly hydrolyzes and turns into the biologically active phenolic form of α-tocopherol [30,31,32].

## 3. Vitamins, Provitamins, and Derivatives

### 3.1. Ascorbic Acid (Vitamin C) as the Major Water-Soluble Vitamin in Milk

Ascorbic acid is the γ-lactone of 2,3-dehydrogulonic acid (classified by its chemical structure [9,21,27,28,29,30]). It can be synthesized endogenously by most animals, whereas this is not the case for humans and most primates (as well as some birds, fish, and guinea pigs), which must obtain it from food [32,33,34,35,36]. This is why, in human physiology and biochemistry, it is named “vitamin C” [2,30]. Regardless of its endogenous or exogenous source, ascorbic acid is an important antioxidant in the biological fluids of animals and humans (blood plasma, lymph fluids, and milk) [30]. The antioxidant activity (AOA) of ascorbic acid is determined by its oxidation–reduction properties, since its molecule contains two enol groups, which represent a donor–acceptor system that can donate or accept two protons [2,30]. Ascorbic acid interacts with many “reactive oxygen species” by donating two protons and converting to dehydroascorbic acid (Equation (1)) [9,21,31].

Ascorbic acid ↔ Dehydroascorbic acid + 2 H^+^ (1)

In order to recover ascorbic acid, two protons can be taken from glutathione (GSH), which converts into oxidized dimer (GSSG, Equation (2)) [21,22,23,24,28,29,30,31,32]:

Dehydroascorbic acid + 2 GSH ↔ ascorbic acid + GSSG(2)

The oxidation of ascorbic acid depends on temperature, light, oxygen, and the number of catalysts [30,31,32,37,38,39,40]. In milk, ascorbic acid is one of the most powerful water-soluble antioxidants (at least among water-soluble vitamins) due to the oxidation–reduction potential of about 330 mV [31]. Its content in cow’s milk is quite high {0.55–3.50 mg/(100 g milk)} according to Russian reference books [30,32]. This range is a little bit smaller according to FAO data {0.0–2.0 mg/(100 g milk) [33]} and its average amount of 1.0 mg/(100 g milk) [33].

According to data from earlier international publications [34,35,36,37,38,39], the range of variations in the content of ascorbic acid in cow’s milk is even larger, being from 5.9 mg/L to 27 mg/L [34,35,36]. By converting these data to the “FAO standard”, these values can be in the range of 0.59–2.70 mg/(100 g of milk) depending on a number of environmental conditions, feeding and maintenance, and the breed and physiology of the animals in the experiments [34,35,36]. For example, in the works of Andersson and Öste [34,35] it was found that the concentration of ascorbic acid in raw milk changes significantly when collected from cows in August (27 mg/L), in March (20 mg/L), or in October (12 mg/L) [34,35]. Such large variations (i.e., by around two times) in the content of ascorbic acid in cow’s milk depending on the season (in connection with feeding features) are confirmed by the data of some other authors [2,8,9,10,11,21]. Interesting data on the content of oxidized and reduced forms of ascorbic acid in milk (after various types of heat treatment) have been presented by several researchers [21,36,37,38,39,40,41]. For example, for “Swedish” cattle, the total content of vitamin C is about 11.6 mg/(kg of milk), of which 10.2 mg/kg belongs to ascorbic acid and 1.4 mg/kg to dehydroascorbic acid [21]. For dairy cattle in Croatia, the concentration of ascorbic acid in raw milk is 5.9 mg/L, whereas the dehydroascorbic acid-only concentration is 1.5 mg/L [36]. It is important to highlight the following data. (A) If the concentration of ascorbic acid in milk is 4.0 mg/L, then in “ultra-high temperature” (UHT) pasteurized milk “with indirect heating”, it is 0.1–1.1 mg/L; however, in “UHT milk with direct heating”, it is 3.9–4.2 mg/L [36]. (B) If the concentration of ascorbic acid in such UHT pasteurized milk is about 0.4 mg/L (i.e., 10 times lower), then the both types of “heating treatment” give almost the same concentration of ascorbic acid (in the range of 0.1–0.2 or 0.3 mg/L) [36]. In our opinion, in the latter case (with a low ascorbic acid content in milk), the data presented are not reliable, although the general trend towards preserving ascorbic acid in “UHT milk with direct heating” is evident.

Moreover, Pizzoferrato [37] found that the retention of ascorbic acid activity was higher after pasteurization at 80–85 °C than at 72–75 °C or 90 °C, and that UHT treatment at 140 °C resulted in lower values than traditional pasteurization [37]. Vahcic et al. [38] found that the ascorbic acid concentration in raw milk was about 14 mg/L; in pasteurized milk, it was 9 mg/L, and in sterilized milk, it was 6 mg/L [38].

In a recent article [41], the content of ascorbic acid in raw cow’s milk changed significantly (from 3.57 mg/kg to 11.6 mg/kg) mainly due to the predominance of succulent feed in the animal’s diet in summer [41]. It was found that using a low-temperature pasteurization regime (65 °C, 30 min.), the content of ascorbic acid decreased within 20–59%, although for a higher temperature regime (76 °C, 5 min.), this was within 18–41% [41]. Similar intervals of “reduction” in ascorbic acid level (activity) in milk during these types of “heating treatment” are presented in the works of some other authors [42,43,44]. All these data [9,21,29,34,35,36,37,38,39,40,41,42,43,44] show the relatively high “rest level” of ascorbic acid in pasteurized milk.

Regarding the effects of storage, the authors of another study found no significant decrease in the ascorbic acid level when pasteurized milk “at low oxygen contents” (prepared at different temperatures [39,40,41]) was stored in a household refrigerator for a few days, but it decreased to half of the initial ascorbic acid value “after 1–2 weeks” [39].

The important positive effects of antioxidants such as vitamin C and its derivatives on the quality and “shelf life” of milk are particularly evident when they are added to milk, especially under “unfavorable storage conditions”. For example, the effect of ascorbyl palmitate (ASCP) added to milk both once and weekly (at an initial ASCP dose of about 100 mg/kg milk fat [45]) is associated with an extension of “shelf life” and a reduction of “unhealthy odor” of the oxidized by-products (mainly “pentanal and heptanol” [42,43,44,45]) during 6 weeks of storage under light at 4 °C [45]. Moreover, ascorbic acid significantly inhibited riboflavin degradation in light-exposed milk, because its antioxidant activity is mainly attributed to the “singlet oxygen scavenging effect” [46]. Thus, ascorbic acid is the most widespread and one of the most active antioxidants in milk among water-soluble vitamins.

### 3.2. Tocopherols (Vitamin E) as the Main Fat-Soluble Vitamins in Milk

**Figure 3:**
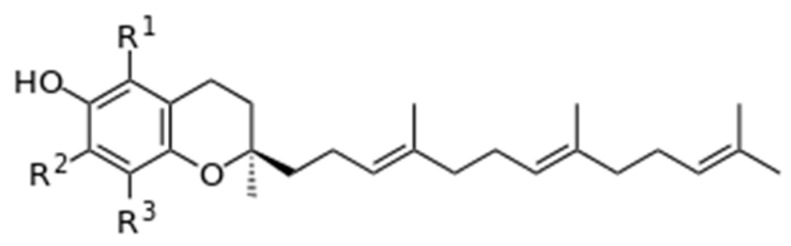
Chemical formula of alpha-tocopherol (5.7,8-trimethyltocopherol), where R_1_ = R_2_ = R_3_ = CH_3_.

Tocopherols are the most widespread chroman derivatives (6-hydroxychromans with isoprene substituents) [21,31], making them an important fat-soluble “vitamin E” [28,29,30]. According to current knowledge, “vitamin E” has eight isomeric forms (“vitamers” [21]), of which four forms have been shown to exhibit antioxidant activity in milk, namely, tocopherols α, β, γ, and δ [18,19,28,29,30] (Figure 3).

Tocopherols effectively eliminate superoxide anion radicals, singlet oxygen, peroxy radicals, OH radicals, ROO radicals, etc., which induce lipid peroxidation (LPO) processes, especially low-density lipid oxidation [31]. The content of vitamin E in cow’s milk is significantly less than vitamin C and varies within fairly wide ranges: 0.4–2.5 μg/mL [27]; 0.20–1.84 mg/kg of milk [28]; 0.02–0.30 mg/100 g of milk [32]; 1.1–1.9 mg/L of milk [40]; or 0.12–0.18 IU of vitamin E (unpublished results from Russian scientists). It is implied that frequently published data on the content of vitamin E (above) include all isomers of tocopherols, but sometimes the level of only α-tocopherol in cow’s milk is measured separately, even without special mention in the papers. For example, it is unclear whether the content of all tocopherols (or only α-tocopherol) in the whole cow milk (or in milk fat) is about 1.2–2.0 μg/g (according to Belarusian scientists [47]); this level significantly exceeds the data of many of other researchers [48,49,50,51,52,53,54,55], as well as the FAO data (0.07–0.08 mg/100 g of milk [33]), which seem to us the most reliable.

Once again, the major tocopherols are distributed unevenly in raw milk, and α-tocopherol definitely presents in the highest concentrations [2,48]. For example, an increased content of α-tocopherol was found in milk fat globule membranes (MFGMs), while γ-tocopherol was found only in cream [49]. This was clearly demonstrated after “intraperitoneal injection of 10 g DL α-tocopherol acetate” [49], which increased the α-tocopherol content in cow milk fat from 13–30 to 50–70 μg α-tocopherol/(g milk fat) on the 2nd and 3rd day after injection [49]. According to the equation proposed by the authors [49], the upper limit of α-tocopherol’s incorporation into MFGM is 97 ± 5 μg/(g MFGM fatty acids). Thus, under normal growing conditions, the α-tocopherol content will be highest in MFGM. However, in creams at α-tocopherol concentrations < 15 μg/(g cream fatty acids), the α-tocopherol level in MFGM will be low, and moreover, it will decrease rapidly. Taking into account the high risks of milk fat oxidation, the samples with the addition of tocopherol derivatives showed higher photooxidative stability [49] and better taste [45,46] compared to samples without such an addition.

A number of researchers [47,50,51,52,53,54,55,56,57,58,59] have determined that the usual amount of vitamin E in fodder for lactating cows is about 1.8 IU (per 1 kg of cow live weight), whereas for dry cows and heifers, it is 2.6 IU (1 IU is taken as the biological activity of 1 mg of α-tocopherol acetate). Moreover, to prevent mastitis and increase the level of vitamin E in colostrum, its share in diets must be increased. The daily amount of vitamin E for cows with high milk yield (of 35–40 kg milk per day) is 1000–1100 IU per head [47], but for cows with lower milk yield, it is 1.5–2.0 times less. During the grazing period, the level of vitamin E in the blood serum of cows varies within 7–20 μg/mL, and during the winter stall period “with full feeding”, this figure is 4–10 μg/mL [47]. In the case of severe vitamin E deficiency, its content in the blood serum of cows drops to 2–2.5 μg/mL, whereas in milk, it falls to 0.6 μg/g (the usual amount is 1.2–2 μg/g) [47].

Bovine milk contains relatively small quantities of tocopherols compared to other animal or human milks [55]. For example, the content of α-tocopherol is about 28–30 μg/100 g in the raw milk of Holstein cows [56], normalized to 3.3% fat in milk [57]. The authors of [56] found that intraperitoneal administration of vitamin E (5 g at the end of the 1st month of lactation) to “dairy cows” is an effective way of increasing the plasma and milk α-tocopherol concentration to 10.9 μg/mL plasma and 1.6 μg/mL fresh milk (after 1 day) from their initial values of 4.5 μg/mL plasma and 0.3 μg/mL, respectively [56]. It is interesting that summer to winter values of α-tocopherol have been documented from 70 μg/100 g to 20 μg/100 g in Finnish milk that has been pasteurized (3.9% fat milk) [58].

Thus, the concentration of these tocopherol derivatives varies both in whole milk and in specific milk structures: α-tocopherol is concentrated in the fat globule membrane, while other tocopherol derivatives are concentrated in cream. Importantly, the concentration of these tocopherol derivatives directly correlates with the fat content of milk that can be useful as a particular marker for animal husbandry.

### 3.3. Some Other Fat-Soluble Vitamins in Bovine Milk

Carotenoids, the main one being retinol (vitamin A), belong to a group of fat-soluble pigments synthesized by a number of plants, seaweeds, and some microorganisms [50,51,52]. Due to space limitations, we will focus here only on vitamin A and β-carotene (its direct precursor), rather than the broader group of carotenoids. The content of retinol (vitamin (A) and β-carotene in cow’s milk varies widely, being 0.2–0.8 μg/mL and 0.2–0.5 μg/mL [27]; 0.2–2.0 and 0.03–0.5 mg/kg [28]; and 0.004–0.10 and 0.002–0.04 mg/(100 g milk) [32]. The same low contents of vitamin A and carotene in cow’s milk (but in narrower ranges of 29–45 μg/(100 g of milk) and 7–23 μg/(100 g of milk)) correspond to the FAO data [33].

It is important to carefully check the absolute values, methods, descriptions, etc., concerning vitamin A or carotene in any, even very solid, publication (especially because of their very low levels in biological samples). For example, the content of vitamin A in cow’s milk is reported to be 46 mg/(100 g of milk), according to the data published by Khan I.T. et al. [9], but this is apparently a typo and, in our opinion, should be read as “46 μg/(100 g of milk)”. The vitamin A content in cow’s milk, according to some other authors, is 0.2–2.0 mg/L [29]; 30–46 μg RE/(100 g of milk) [33]; 0.30–0.45 mg/L [40]; 126 IU/(100 g of milk) or 31 μg RE/(100 g of milk) [55], etc. Such pronounced variations in the content of vitamin A in milk are associated with the following factors: (A) great difficulties in its measurement in any biofluid; B) fast oxidation due to the conjugated “-CH=CH-bonds” in its biochemical structure; and C) quantitative fluctuation even for the same animals depending on feeding and maintenance, seasonal fluctuations, etc.

It is interesting that summer to winter values of total retinol (or β-carotene) vary from 61.9 (31.5) µg/100 g to 41.2 (10.5) µg/100 g in Holstein cow milk [59]. Summarizing data from various publications from the end of the last century up to the beginning of this century, the range of variations in the vitamin A content in cow’s milk can be considered to be 4–65 μg/100 g, depending on a number of environmental conditions and the feeding and maintenance, breed, and physiology of the animal [29,30,31,32,33,34,35,36,37,38,39,40,41,42,43,44,45,46,47,48,49,50,51,52,53,54,55,56,57,58,59].

The other important roles of carotenoids are well known, but their antioxidant activity is the most widespread and still underestimated. Moreover, the presence of some other antioxidants, along with carotenoids, leads to the manifestation of synergism in their actions, especially in the regulation of redox processes, and it is the most important indicator of their biological value [50,51,52]. Thus, a combination of antioxidant vitamins can more effectively stimulate the activity of a number of enzymes in the body’s antioxidant defense, and they can inhibit lipid peroxidation processes induced by transition metals [21,22,23,24,25,50,51,52].

All of these comments can be applicable to vitamin D and K types, although their contents in milk are rather low. Moreover, vitamins D and K are very difficult to analyze (even using modern methods such as HPLC). So, only few reports on their contents in milk are available, without deep insight into their antioxidant function.

The total content of vitamin D and all its hydroxyl metabolites (such as 25-OHD or 25,26-OHD or 1,25-OHD or 24,25-OHD) in cow’s milk varies widely, with 6–25 IU/L [27], 13–105 IU/L [55], and 0.1–0.3 μg/(100 g of milk) being reported. The latter corresponds to the FAO data [33] and is close to the average amount of 1.2 μg/kg [28]. For example, the content of 25-hydroxyvitarnin D metabolite is about 29–99 IU/l or 145–497 ng/l (1 IU is about 5 ng 25-OHD) [55].

In the majority of dairy food samples [55,56,57,58,59,60] measured using the HPLC method in the lab of Dr. Caroline Bolton-Smith (University of Dundee, Dundee, UK) under the existing Royal Society of Chemistry/Ministry of Agriculture, Fisheries and Food (RSC/MAFF) food code, vitamin K content has a “negligible” value range (i.e., <20 µg/kg) [60]. For example, there is 3.6–9.0 (µg/kg) and about 2 µg/kg of phylloquinone (vitamin K_1_) content in full-fat and semi-skimmed milk, respectively [60]. Older studies, as summarized in the famous handbook edited by Jensen R.G. [55], present the vitamin K_1_ content in raw milk at levels from 4.9–8.7 pg/L up to 19.7 pg/L in pooled samples collected over a year from cows of a few breeds (Friesian, Jersey, or Guernsey) [55]. It is difficult to find reliable data on the antioxidant function of vitamin K types in milk, but they must act as another typical phenolic antioxidant in protecting the cellular components (lipids, proteins, and genome elements) from oxidative damage [21].

It is important to highlight the latest recommendations of the RDAs [29] and Russian officials [32] on the vitamin intake of an adult male/female: vitamin D—15/10 (µg/day); vitamin E—15/15 (mg/day); vitamin K—120/120 (µg/day). However, vitamin A is about 100,000 (ME/day) for adults and 20,000 (ME/day) for children.

### 3.4. Some Other Water-Soluble Vitamins in Bovine Milk

The contents of water-soluble vitamins in cow milk are as follows (with average ranges taken from major international and Russian books): thiamine (vitamin B_1_)—0.3–0.5 mg/kg [28], 350–460 µg/L [61], or 0.01–0.08 mg/(100 g of milk) [32]; ribofiavin (vitamin B_2_)—1.0–2.0 mg/kg [28], 1600–1900 µg/L [61], or 0.06–0.30 mg/(100 g of milk)) [32]; niacin (vitamin B_3_)—0.2–2.0 mg/kg [28], 350–460 µg/L, or 0.08–0.24 mg/(100 g of milk) [32]; pantothenic acid (vitamin B_5_)—2.8–5.6 mg/kg [28], 3130–3600 µg/L, or 0.26–0.64 mg/(100 g of milk) [32]; pyridoxine (vitamin B_6_)—0.3–1.5 mg/kg [28], 400–650 µg/L, or 0.26–0.64 mg/(100 g of milk) [32]; folic acid (vitamin B_9_ or B_c_)—0.001–0.006 mg/kg [28], 50–60 µg/L, or 2.6–5.5 µg/(100 g of milk) [32]; cobalamin (vitamin B_12_)—0.002–0.005 mg/kg [28], 3–5 µg/L [61], or 0.1–0.3 µg/(100 g of milk) [32]; biotin (vitamin H)—0.02–0.06 mg/kg [28], 20–47 µg/L [61], or 2–5 µg/(100 g of milk) [32].

Most thiamine molecules, as well as the other water-soluble vitamins found in milk, are produced by microorganisms in the rumen [32,61]. This is why there is almost no “vitamin B” deficiency in cattle. An important issue is thiamine content in cow’s milk, which is small compared to “vitamin C” and is about 0.55–3.50 mg/(100 g milk), according to Russian reference books [2,32], which is close to the FAO data, which is on average 1.0 mg/(100 g milk) or in the range of 0.1–2.0 mg/(100 g milk) [33].

Pantothenic acid and biotin occur in milk in the free forms. In contrast, the major chemical form of folic acid in cow milk is as 5-methyltetrahydrofolic acid [61]. It is important to highlight that the majority of the water-soluble vitamins can have some derivatives or transport forms, as well as the “active” (coenzyme) forms for enzyme usage. For example, the predominant form is hydroxycobalamin, with minor amounts of methyl- and adenosynlcobalamins in cow milk [61] Thiamine occurs in its free (50–70%), phosphorylated (18–45%), and protein-bound form (5–17%) [62]. The major types of “flavin forms” are riboflavin, at 60.5%; flavin adenine dinucleotide, at 25.6% (coenzyme form); the hyroxylethyl form, at 11%; and traces of three other derivatives. It is important to highlight that the hydroxyethyl derivative is a potential antivitamin. Riboflavin is not affected by pasteurization but is photodegradable when exposed to sunlight in clear containers [62]. The vitamin B_6_ activity in raw cow milk is partitioned into pyriodoxal (80%); pyridoxamine (20%), and traces of pyridoxamine phosphate [62]. Vitamin B_6_ is not affected by pasteurization treatments (<10% loss) but is photodegradable [61]. Destruction of riboflavin catalyzes the photochemical oxidation and loss of ascorbic acid. The latest recommendations of the RDAs [29] (or Russian officials [32]) for the vitamin intake of an adult male (mg/day) are as follows: thiamine (vitamin B_1_)—1.5 (1.5); ribofiavin (vitamin B_2_)—1.8 (1.7); niacin or equivalent (vitamin B_3_)—2.0 (1.9); pantothenic acid (vitamin B_5_)—6.0 (6.0); pyridoxine (vitamin B_6_)—2.0 (2.0); folic acid (vitamin B_9_ or B_c_)—0.2 (0.2); cobalamin (vitamin B_12_)—0.003 (0.002); and biotin (vitamin H)—0.05 (0.002). Although influence of pasteurization on these vitamins is known to a great extent, it is still necessary to systematically study the effects of further handling, types of packaging, length of storage, and storage temperatures, particularly with modern analytical methods. In general, under the same storage conditions, “low-fat” and “non-fat” milks will contain about the same amounts of these water-soluble vitamins as whole (raw) milk [61].

### 3.5. Vitamin Requirements of Dairy Cows and Pathologies Associated with Vitamin Deficiency

Intensification of animal husbandry [63,64,65] requires a complete balance of animal feeding not only with the main organic and mineral compounds [66,67,68,69] but also with vitamins that play an important role in animal metabolism [28,29,30,31,32,56,61]. This is especially important for lactating cows, which require increased inclusion of a number of vitamin supplements in their feed [70,71,72]. Since most of the water-soluble vitamins in cows are produced by microorganisms in the rumen [32,61], the need for vitamins such as A, E, and D predominates [70]. For example, for six groups of cows (live weight 500–600 kg) producing 18–20/20–24/26–28/30–32/34–36/38–40 L of milk per day, the need for carotene is 610–680/710–785/825–895/1000–1125/1180–1255/1320–1385 mg per head per day [70], respectively. In addition to the direct effect of vitamins supplied in the form of feed additives [70,71,72] on animal physiology and biochemistry, a number of studies suggest their effect on the general immune system (including “the activation of cells involved in tumor immunity” [73]) and on the “regulation of the expression of a number of genes” [74], including those affecting animal productivity and the biological safety of raw materials and products of animal origin [75,76,77,78,79]. Moreover, various issues of vitamin bioavailability [80,81,82] and comparison of natural and synthetic antioxidants, etc. are presented in detail in papers [83,84,85,86,87,88,89]; therefore, they will not be considered in full here.

There are numerous data [9,21,29] on relationship of vitamin deficiencies with corresponding diseases in animals (partly initiated by oxidative stress, ROS, etc.), e.g., vitamin A deficiency may be accompanied by night blindness, impaired resistance to infectious diseases, less body growth, etc. [90,91,92]; vitamin group B deficiency may be associated with stunted growth, loss of appetite, indigestion, etc. [93,94,95]; and vitamin C deficiency may be associated with fatigue, pyorrhoea, susceptibility to infection (scurvy), etc. [96,97,98,99]. It is important to highlight the data [9,21,29,34,35,36,37,38,39,40] concerning the relatively high “rest level” of almost all vitamins in pasteurized milk, but not in sterilized milk.

## 4. Milk Proteins, Carbohydrates, and Lipids

At first glance, milk proteins can be divided into three groups: (1) caseins (about 4/5 of all milk proteins with moderate antioxidant activity); (2) whey proteins (structurally the most diverse group, possessing the highest antioxidant activity of the three groups); and (3) proteins of the membranes of fat globules, which make up only about 1% of all milk proteins and have almost no antioxidant activity (therefore, they will not be discussed separately) [100,101,102,103].

### 4.1. Caseins 

Caseins (19–25 kDa) are the most abundant (80 ± 5% [2,3,4,9,21,22,23,24,28,29,30]) fraction of milk proteins [103,104,105]. In absolute terms, caseins constitute 2.2–3.0 g/100 g from 3.05–3.85 g/100 g of “total protein” (TP) according to Russian sources [30,32] or 26–30 g/kg from 32–36 g/kg TP according to a number of international sources [9,21,28,106,107,108,109]. Due to the difference in the content of amino acids and phosphate residues in milk, a number of casein fractions are distinguished, the most prominent of which is the α-fraction [108,109,110]. Moreover, at present, α_s1_-casein and α_s2_-casein can be isolated; their contents in milk are about 10.0–11.9 and 2.6–3.1 g/kg (appr. 31 and 8% of total protein, w/w), respectively [21,28]. Very important and quite numerous are the fractions of β-casein (including γ-casein) and κ-casein, the contents of which in milk are about 9.9–11.9 and 3.3–3.5 g/kg (or 31 and 10% of total protein, w/w), respectively [110,111,112]. Thus, these main casein fractions (α-, β- and κ-fractions) are presented in milk in the form of macromolecular aggregates or supramolecular “casein micelles”, whose size is approximately 200–400 nm, with the diameters of individual “casein micelles” (or so-called “submicelles”) being about 10–15 nm [28,29,30,32,112,113,114,115]. The concentration of “casein micelles” is about one thousand per 1 mL of milk. In the opinion of many researchers, the antioxidant activity of proteins is due to their functional groups (primarily sulfur-containing groups) [28,29,30,32], although some authors believe that in the case of casein micelles, it is due to phosphates [28,29,30,32]. For example, the phosphate content in α-, β-, and κ-caseins is about 8–10, 4–5, and 1 mole per mole of casein, respectively [28,29,30,32]. It is known that methionine is presented in all three fractions, whereas cysteine is mainly presented in the α- and β-caseins only. If we consider that α- and β-caseins are located inside the “submicelles” and κ-caseins are outside, then the low antioxidant activity of “casein micelles” becomes understandable [104,105,106,112,113,114,115].

The author believes that the main contribution to the antioxidant activity of milk caseins is made by the side groups of only a few selected amino acid residues (primarily aromatic rings [28,29,30,32]), which will be discussed in detail in the section below (describing amino acids). It is worth mentioning here that the antioxidant activity of casein molecules may be associated with the ability of phosphoserine residues to form complexes with non-heme iron to absorb ROS [28,29,30,32]. Caseins are also capable of inhibiting both lipid oxidation and autooxidation catalyzed by some enzymes [6,8,18]. The generalized structure of supramolecular “casein micelles” (stabilized by phosphates and calcium cations) and their “individual” components is presented in great detail in many books and reviews [28,29,30,32,101,102,103,104,105,106,107,108,109,110] and will therefore not be discussed here. The author believes that the major antioxidant activity of milk proteins is not due to caseins but to a greater extent to the antioxidant properties of whey proteins (although the content of the latter—whey proteins—in milk is only about 19 ± 0.5% [28,29,30,32,111,112,113,114,115,116,117,118,119].

### 4.2. Whey Proteins

A large group of biologically active substances in milk are represented by whey proteins, i.e., α-lactalbumin, β-lactoglobulin, γ-globulins (immunoglobulins), serum albumin, etc. [2,9,19,28,29,30]. In addition, this group includes lactoferrin and some other so-called “minor” proteins [116,117,118,119,120,121,122], which will be described below. The antioxidant activity of milk proteins is due, first of all, to particular proteins that are capable of chelating iron and other cations; it is also due to the binding of free radicals by the sulfur-containing groups of amino acid residues [2,3,4,9,21,22,23,24,28,29,30]. The aforementioned group of whey proteins includes the main enzymes of milk (as part of the general antioxidant system), which differ in the principle of their action: some prevent the formation of radicals or neutralize their action [7,8,9,21], while others catalyze the synthesis or regeneration of non-enzymatic antioxidants [19,21]. Only the individual proteins and protein fractions with relatively high antioxidant activity will be considered below.

#### 4.2.1. Lactoferrin

Lactoferrin (LF) is an iron-binding glycoprotein (80 kDa, 696 AAs, [2,3,4,9,21,22,23,24]) that presents in cow’s milk at a concentration of approximately 0.1 g/L [2,3,4,9,21,22,23,24,123]. Lactoferrin performs several important biochemical functions, including those related to its antioxidant activity, such as iron absorption and transport; bacteriostatic and bactericidal action; and functioning as a growth factor [9,14,15]. It is reported [123] that iron binding by LF inhibits the conversion of hydrogen peroxide to hydroxyl radicals. The antioxidant properties of lactoferrin are also manifested in its ability to bind lipopolysaccharides, thereby limiting the formation of free radicals [13]. In addition, lactoferrin activates some other enzymes of the antioxidant system [14,17].

A number of studies have described the structural peculiarities and various factors that affect the relative LF concentration in milk [123,124,125,126,127,128,129]. For example, the LF concentration in milk has consistently been associated with the following metrics: somatic cell count (SCC), BSA level, stage of lactation, and milk production [130,131,132,133,134,135,136,137]. Interestingly, the LF content in the case of “multiparous” cows has been found to be about 2–3 times higher than that of “primiparous” cows (in first-calf heifers) [130]. These findings suggest that milk LF may be useful as an indicator for some infections in dairy cows [130,131,132,133,134,135,136,137,138].

#### 4.2.2. Catalase

Catalase is a large protein (more than 200 kDa) and one of the most prominent representatives of the oxidoreductases (EC 1.11.1.6), containing four heme-bound iron ions in the active site of each enzyme molecule [2,3,4,28,29,30]. Catalase oxidizes the substrate, hydrogen peroxide (H_2_O_2_), resulting in the formation of water and molecular oxygen (Equation (3)) [138,139,140].

H_2_O_2_ + H_2_O_2_ → O_2_ + 2 H_2_O (3)

Catalase has a very high capacity to destroy H_2_O_2_ (more than 40 thousand molecules decomposed per minute per enzyme molecule), and from this point of view, catalase is one of the most active known enzymes [28,29,30,138,139,140]. Unfortunately, very few studies have been devoted to the study of catalase’s activity in cow’s milk [141,142,143]. For example, in [143], significant changes in catalase activity were found (from 0.44 relative units (in the middle of lactation) to 1.41 relative units (at the beginning and end of lactation) in different groups of 280 animals). During the period of milk production and lactation decline (groups 1 and 6), strong positive correlation coefficients (0.51 and 0.57) between catalase and fat were observed [143]. In contrast, catalase activity in other biological samples of animal, plant, and microbial origin has been studied in a greater number of cases [144,145,146], and a high capacity to degrade H_2_O_2_ has been observed (k_cat_ = 3.8 × 10^7^ s−^1^, with a Michaelis constant of 1.1 M) [144]. Currently, catalase is being considered as a therapeutic agent for a number of diseases [144,145,146,147] associated with ROS formation [138,139,140,141]. In particular, catalase’s efficiency as an antioxidant has been demonstrated in animal models of liver ischemia/reperfusion injury, chemical tissue damage, and tumor metastases to the liver, lungs, and peritoneal organs [146].

Catalase’s applications range from direct supplementation in conditions characterized by oxidative stress to gene therapy approaches to enhance endogenous catalase activity [144,145,146,147]. In the development of this topic [144,145,146,147], one very important review concerns the regulation of a number of non-infectious diseases through the combined use of antioxidants and enzymes [148]. It has been shown that the interaction of some antioxidants (flavonoids, phenolic derivatives, quercetin, vitamin C, etc.) with an enzyme (like catalase) reduces the number of free radicals, therefore reducing oxidative stress [148]. In this context, young scientists from the Orel State Agrarian University [149] studied catalase activity (“permanganometric” method) in the milk of cows with clinical and subclinical mastitis [149]. This study confirms that catalase activity increases sharply (by almost 10-fold) in the milk of cows not only with mastitis but also with its subclinical form [149]. Since mastitis causes multimillion-dollar losses in the dairy industry, this study [149] confirms the usefulness of this method for the timely diagnosis and monitoring of treatment of diseased animals. A “polarographic” method showed that the average catalase activity in cow’s milk was 1.95 U/mL, which is approximately ten times lower than that in human milk [150]. Catalase in milk usually works actively in combination with superoxide dismutase, about which there is a large pool of literature, as detailed below [151,152,153,154,155,156,157,158,159].

#### 4.2.3. Superoxide Dismutase

Superoxide dismutase (SOD, EC 1.15.1.1.) refers to a group of metalloenzymes with antioxidant activity; it has a molecular weight of approximately 33.2 kDa [151,152,153] and consists of two identical subunits, each of which contains one copper and one zinc ion; a disulfide bridge within the chain; one sulfhydryl group; and an acetylated terminal amino group [151,152]. It is one of the main enzymes of the antioxidant system, catalyzing the dismutation reaction of superoxide anion radicals (maintaining their concentration in the cell at a low level) and reducing the likelihood of the formation of even more active singlet oxygen [151]. Depending on the metal ion in the active center of the enzyme, several SOD isoenzymes are distinguished, among which Cu-SOD + Zn-SOD has the highest activity [152]. SOD is inhibited by cyanide and inactivated by hydrogen peroxide [151,152]. Iron ions block the secretion of the enzyme [151,152,153]. The extracellular form of SOD [154] is present mainly in extracellular spaces, but it has been found in the lungs, heart, kidneys, and placenta. It also has a high affinity for certain glycosaminoglycans (such as heparin and heparin sulfate) [153,154,155]. The effectiveness of superoxide dismutase preparations in the treatment of a number of diseases has been demonstrated experimentally [156,157,158,159]. Interestingly, as in the case of catalase, the interaction of SOD with some antioxidants (flavonoids, phenolic derivatives, quercetin, vitamin C, etc.) enhanced its ability to reduce the number of free radicals (and therefore to reduce oxidative stress) [148].

#### 4.2.4. Glutathione Peroxidase

Glutathione peroxidase (GSHPx, EC 1.11.1.9) is a selenium-encompassing enzyme that provides the most effective protection against lipid peroxidation (LPO) [160,161,162,163,164,165]. Glutathione peroxidase exists in milk in a complex form within the casein fraction [6,20]. It catalyzes the breakdown of H_2_O_2_ and organic hydroperoxides (R-OOH) by glutathione (γGlu-Cys-Gly) following a chemical reaction [160,161,162,163,164,165]. More than 90% of GSHPx exists in milk as an extracellular enzyme, and it is the only enzyme that effectively fixes selenium (about 30% of the total Se). Its Se group is oxidized by peroxide and then reduced by glutathione, which is converted into oxidized glutathione. Selenium can be incorporated specifically into the amino acid chain of proteins as “selenocysteine” [6,7] and even as “selenomethionine”, becoming a part of the active center of glutathione peroxidase. The concentration of GSHPx in cow milk ranges from 12 to 30 U/mL, which is very low compared to that in human milk [160,161,162,163,164,165]. Its concentration varies among mammals, and its activity is mainly dependent on the concentration of selenium [161,162,163,164]. Antioxidant activity and selenium content decrease with the progression of lactation [161,162,163,164]. For example, in [166], high correlations were established between catalase (glutathione peroxidase) activity and the total number of somatic cells (with the number of neutrophils). Since both of these enzymes (catalase and glutathione peroxidase) are closely associated with oxidation–reduction processes in all cells, changes in their activity are one of the markers of “oxidative stress” in cells and the animal’s body as a whole [9]. All of the listed parameters are important for assessing the antioxidant component of milk and its further processing [160,161,162,163,164,165,166].

#### 4.2.5. Lactoperoxidase

Lactoperoxidase (LPO, E.C. 1.11.1.7) is a complex enzyme system (called the “lactoperoxidase system” or LPOS) present in milk that performs a protective function by inhibiting bacterial growth [167,168,169]. LPOS is synthesized by mammary gland cells, and some of it enters milk with leukocytes. Peroxidase is present in milk in amounts ranging from 3 to 10 mg%. The LPOS content of bovine milk is about 30 mg/L [170,171] or 2–4 mg% [29] (i.e., in significantly greater quantities compared to other milk enzymes), whereas human whey contains only about 0.77 mg/L LPOS [172]. Lactoperoxidase is the second most abundant enzyme in milk, and its main role is to protect the mammary gland and intestine of infants from bacterial infections. It can inhibit the growth and metabolism of various types of microorganisms [173,174,175]. The components of the lactoperoxidase system are the following: (1) milk lactoperoxidase itself (LPOS); (2) thiocyanate (SCN^−^) or iodite (I−), as an oxidizable substrate present in milk; and (3) hydrogen peroxide (H_2_O_2_) as an additional oxidizing agent used to enhance the activity of the system. The mechanism of action of lactoperoxidase is associated with the oxidation of thiocyanate ions (in the presence of hydrogen peroxide) to hypothiocyanate (OSCN^−^) or hypoiodite (OI−), which have a broad spectrum of antimicrobial properties (suppressing the growth of bacteria, fungi, and viruses), and it increases the shelf life of milk [173,174,175].

The advantages of LPOS are several. It has the ability to preserve raw milk at ambient temperature, making it a valuable tool in regions with limited access to refrigeration, and it is a cheaper and more accessible alternative to refrigeration for small-scale dairy producers, improving the “quality, safety, and marketability of milk” (without the need for “aggressive chemical additives”). The LPOS system is effective against a wide range of microorganisms, including pathogenic bacteria, mold, and yeast [170,171,172,173].

Its limitations are as follows. (1) For the system to function effectively, the presence and coordinated operation of all three components (LPO, SCN^−^, and H_2_O_2_); (2) bacteriostatic action involves inhibition of growth but not destruction of all microbes; and (3) the effectiveness of LPOS depends on the initial bacterial load and the temperature of the milk [173,174,175]. LPOS activity is used to evaluate the effectiveness of pasteurization, as when milk is heated to temperatures above 75–80 °C, lactoperoxidase is destroyed, which is confirmed by the absence of blue coloration in special tests [173,174,175]. LPOS has great potential to be used in various areas such as preservation and shelf life elongation of milk, milk products, meat, and plants, including fruits and vegetables, oral care, and diagnosis, immunomodulation, and treatment of nephrotoxicity. [173,174,175].

#### 4.2.6. Ceruloplasmin

Ceruloplasmin (CP, EC 1.16.3.1) is one of the conserved proteins of a large “family” of metalloproteins with enzymatic properties. The key function of CP is to regulate iron transport for incorporation into cells and essential iron-containing proteins [176]. Together with other “multimedrocopper ferroxidases”, ceruloplasmin maintains iron homeostasis in mammals. Each of these enzymes contains at least six copper atoms and acts as an intermediate electron acceptor [176,177,178]. Ceruloplasmin is synthesized by the liver, mammary gland, and other cells, and CP is found in both secreted and membrane-bound forms [177]. The detailed structure of ceruloplasmin and its role in iron metabolism have been demonstrated in a number of fundamental studies [177,178,179]. For example, in the works of Vashchenko G. [178] and Moshkov K.A. [179], a special role was noted in the copper-binding centers of proteins of this family of two histidine residues (HISs), one methionine (MET), and one cysteine (CYS). Moreover, the most significant role in the coordination of copper ions (Cu^2+^), of which there are at least six per molecule, is assigned to histidine residues [179]. Ceruloplasmin is found in blood serum, milk, cerebrospinal fluid, and other extracellular secretions [180]. In lactating mammals, up to 30% of the digestible copper bypasses the liver and is absorbed by mammary gland cells, where the level of ceruloplasmin gene expression is increased. For newborns, ceruloplasmin is a source of copper, and the molecular genetic mechanisms responsible for copper homeostasis are highly adapted to milk CP [180]. According to [6], milk contains 65.37 to 89.85 μg/L of copper. In [181], the amount of ceruloplasmin in milk obtained from healthy cows in the control group was found to be 0.73–2.11 units/(g protein), whereas in animals with various types of bacterial contamination in milk, the CP level ranged from 3.35 to 8.02 units/(g protein) [181].

For industrial dairy production, it is important not only to assess the quality of raw milk but also to preserve it, preventing premature oxidation of milk fat. In this regard, ceruloplasmin is of interest as a protein indicator of the acute phase of inflammation; it also acts as a link in the antioxidant defense system. These properties of ceruloplasmin may be useful for maintaining proper product quality. The role of ceruloplasmin in the coordination of copper has been described above, and as is known, excess copper in milk is associated with the risk of developing oxidative processes, the appearance of a specific taste, and an increased risk of product spoilage [182]. On a global scale, attention is largely focused on the role of CP as an indicator of mastitis pathology [183,184,185]. The content of ceruloplasmin and copper ions (in the blood) undergoes a physiological increase immediately after calving, which can be easily explained by its function as a heat shock protein, which increases in the acute phase of inflammation [184]. In the works of Szczubial M. [185] and Saleh N. [186], it was shown that an increase in the level of ceruloplasmin activity in cows’ milk coincides with the manifestations of both clinical mastitis (of varying severity) and subclinical mastitis, which was noted in the work of Sadat A. [187]. Measurement of milk CP is in most cases carried out using a modified “Revin method”, based on the enzymatic reaction of CP in the oxidation of p-phenylenediamine, which is stopped by the addition of sodium fluoride [188].

Research by the group headed by the author of this review focused on the study of CP in connection with the level of copper in the milk of healthy cows at different stages of lactation [189]. High correlation coefficients between CP and copper were established, but the direction of these correlations may be negative (with copper content greater than 80 μg/L) or positive (with copper content less than 80 μg/L) [189]. The average values of ceruloplasmin were about 0.44–0.49 mg/mL, and those of copper were 71–83 μg/l in the milk of four groups of healthy cows (from 1–2 to 8–9 months of lactation) [189]. The specified intervals of ceruloplasmin and copper content can serve as important guidelines in determining “reference intervals” for the raw milk of cows at different stages of lactation.

#### 4.2.7. Xanthine Oxidase

Xanthine oxidase (XOD, EC 1.17.3.2) is a complex molybdenum–flavoenzyme complex secreted by mammary gland cells [190,191,192,193]. XOD is the major protein component of fat globule membranes and is located on their inner surface [190,191,192]. Fat globule membranes contain approximately 80% of the total xanthine oxidase in raw milk, although they account for only about 20% of the total protein in the MFGM [190,191]. Xanthine oxidase oxidizes various aldehydes and purine bases (xanthine, etc.) to the corresponding acids and can also reduce nitrates to nitrites [190,191,192,193]. The use of synthetic drugs such as allopurinol or febuxostat may induce a wide range of side effects, including nausea, diarrhea, arthralgia, headache, increased hepatic serum enzyme levels, and rash [193,194,195].

#### 4.2.8. Sulfhydryl Oxidases

Sulfhydryl oxidases (SOXs, 89 kDa, EC 1.8.3.2.) are divided into different families and can be either intracellular or secreted enzymes [196]. All of these enzymes generate ROS rather than degrade them because they catalyze the formation of disulfide bonds by oxidizing free thiol groups (RSH) in various thiol-containing molecules, using molecular oxygen (O_2_) as an electron acceptor and forming hydrogen peroxide (H_2_O_2_) as a byproduct [196,197,198,199].

2 RSH + O_2_ → RSSR + H_2_O_2_ (4)

On the other hand, these enzymes are vital for all organisms, since disulfide bonds play a key role in the folding of synthesized proteins. Furthermore, SOXs are important for the oxidation of reduced glutathione and the regeneration of denatured proteins [30].

Recently, the existence of at least two different types of coenzymes (both metals and flavins) capable of incorporating into SOXs has been demonstrated [196]. Unfortunately, there is insufficient space to describe metal-dependent oxidases from the skin, kidneys, and other tissues [196,197,198]. It is worth mentioning that a series of studies were conducted on the isolation and characterization of SOXs from cow’s milk, as summarized in article [199]. This enzyme contained 0.5 equivalents of iron per subunit and was completely inhibited by 1 mM EDTA, although activity was restored by the addition of iron or, to a lesser extent, divalent copper and manganese [199].

Most sulfhydryl oxidase (about 55%) is found in the globulin fraction; about 23% of the enzyme is bound to fat globule membranes, and about 21% is bound to casein [2]. Sulfhydryl oxidase activity is about 18,600 and 14,300 U in the skim and whey parts of cow’s milk, where the total protein concentrations are 33 and 9 mg/mL, respectively [200].

### 4.3. Milk Peptides and Amino Acids

A large number of biologically active peptides found in raw milk and formed after its technological processing or after hydrolysis of milk proteins in the gastrointestinal tract have been described in the literature [28,29,30,201,202,203,204,205,206].

#### 4.3.1. Peptides with More than Six Amino Acids

A number of milk peptides possess antioxidant properties [2,3,4,28,29,30,190]. The antioxidant activity of peptides depends on their amino acid structure and, consequently, on the specificity of the proteases involved in the hydrolysis process. Hydrolyzed milk proteins (especially caseins) often contain residues of proline, histidine, tyrosine, and other amino acids [207,208,209]. For example, from casein proteins, under the action of trypsin, an octapeptide of the sequence VAL-LYS-GLU-ALA-MET-ALA-PRO-LYS is formed, which is capable of inhibiting the process of lipid peroxidation, occurring both enzymatically and non-enzymatically, and in the “superoxide anion scavenging activity” (SOSA) [207]. From these same proteins, but under the action of pepsin, a hexapeptide TYR-PHE-TYR-PRO-GLU-LEU is formed, which is involved in the neutralization of various types of radicals [208]. The same properties (“radical scavenging activity”) are possessed by the polypeptide TRP-TYR-SER-LEU-ALA-MET-ALA-ALA-SER-ASP-ILE-TRP-TYR-SER-LEU-ALA-MET-ALA-ALA-SER-ASP-ILE-TYR-VAL-GLU-GLU-LEU, which is formed from β-lactoglobulin under the action of a number of endopeptidase-type enzymes [209]. It is known that the antioxidant potential of milk peptides can increase due to the content of vitamin-based antioxidants in milk, especially in the presence of vitamins C and E [207,208,209]. Interestingly, in article [210], in addition to the above-mentioned and similar peptides showing moderate free radical scavenging activity in ABTS assays, they demonstrated activation of the “Keap1-Nrf2” system [210]. This activation ensures translocation of the transcription factor Nrf2 from the cytosol to the nucleus, which triggers overexpression of some antioxidant enzymes [210]. Thus, milk bioactive peptides are important nutritional factors that perform multiple functions and have various properties, including antioxidant, antihypertensive, anticancer, and antimicrobial. Hereafter, we will describe only the most important and thoroughly studied tripeptide (L-gamma-glutamyl-L-cysteinyl-glycine) or “glutathione”, the biosynthesis and catabolism of which are described by the so-called glutamyl cycle [30,165,166,190,211,212,213,214,215].

#### 4.3.2. Tripeptide Glutathione

Glutathione (GSH) in its reduced form can function as an antioxidant in many ways [211,212,213,214,215], among which are chemically interacting with singlet oxygen, superoxide, and hydroxyl radicals or directly destroying free radicals, and stabilizing membrane structure by transporting acyl peroxides formed through lipid peroxidation (LPO). Reduced glutathione acts as a hydrogen ion donor in many reactions. During oxidation, two molecules form a dimer via a disulfide bond, which is the oxidized form of glutathione:

2GSH → GSSG + 2 H^+^ (5)

The reverse reaction is catalyzed by the enzyme glutathione reductase (GR) using the NADP-H(+H^+^) cofactor:

GSSG + NADP-H(+H^+^) → 2GSH + NADP^+^ (6)

GSH is a coenzyme for several enzymes whose activity is based on changes in the redox potential of glutathione. Reduced glutathione (GSH), a low-molecular-weight thiol, is the predominant form (90–95%) in many animal cells. According to Meister A.’s data collected in a review [165], the molar concentration of GSH in cells is 1–10 mM, which is significantly higher than the concentration of most similar bioorganic substances. In addition, glutathione is necessary for maintaining the reactions of the ascorbate–glutathione cycle associated with the neutralization of hydrogen peroxide [165]. Oxidized glutathione (GSSG) is a low-molecular-weight thiol found in all types of cells and in the extracellular space [165]. The content of GSSG in and outside cells is low and tightly regulated relative to its conjugated compound, amounting to 10^−4^–10^−5^ M versus 1–10 mM GSH [165]. The role of oxidized glutathione in physiological processes is considered primarily in terms of cellular reactions of glutathione [211,212,213,214,215]. In an important study [205], glutathione was detected in collected whey fractions using a dedicated kit, which showed that the amount of glutathione was 2.915 mg in 100 mg of prepared lyophilized whey sample [215]. In the same study [215], its potential as an anticancer agent was investigated by evaluating the cytotoxic effect of purified serum glutathione on the HePG2 cell line in vitro at different concentrations and using different treatment times. The positive effect of glutathione was assessed using the MTT (3-(4,5-Dimethylthiazol-2-yl)-2,5-diphenyltetrazolium bromide) assay. A significant and reliable (*p* < 0.05) cytotoxic effect was found for all experimental conditions. The greatest growth inhibition (90%) was observed at a glutathione concentration of 400 μg/mL after 72 h of exposure [215].

Living cells use three lines enzymatic protection against active oxygen compounds, including superoxide dismutase, catalase and glutathione peroxidase; glutathione peroxidase; and glutathione transferase. These three lines of defense sequentially reduce superoxide radicals, H_2_O_2_, and organic hydroperoxides. A fourth line of defense is also distinguished; it is the neutralization of secondary products of peroxidation of other oxidized compounds, in which glutathione transferase, glyoxylase, and formaldehyde dehydrogenase participate. Obviously, glutathione is involved in three of the four lines of defense and, therefore, makes a major contribution to the functioning of the antioxidant system. Glutathione, glutathione peroxidase, glutathione transferase, glutathione reductase, and NADP-H form the glutathione antioxidant system, in which glutathione reductase and NADP-H are necessary for the reduction of oxidized glutathione and its recycling. Reduction of hydroperoxides by glutathione peroxidase and glutathione transferase prevents the progression of peroxidation and the formation of its secondary metabolites. Glutathione transferases play a key role in the detoxification of secondary peroxidation products and other oxidized substances. Thus, the complex glutathione system effectively protects cells from oxidative stress [215].

#### 4.3.3. Milk Amino Acids

According to the author of [2,30], amino acid residues in proteins and peptides make a significant contribution to the antioxidant system of milk, since the content of the latter in milk is significantly higher than that of classical antioxidants [2,20,28,29,30,31,32]. Moreover, this function of amino acids (both free and contained in proteins) is associated with their side chains [216]. Although the antioxidant activity of an individual amino acid is small, given the high protein concentration in milk, one can be confident that their combined effect will be significant [216]. In Table 2, the author lists the most representative data [2,20,28,29,30,31,32] on the amino acid content of milk proteins.

According to data from various authors [2,20,28,29,30,31,32], as summarized in Table 2, the range of values for each specific amino acid is quite wide, since it depends on many physiological and biochemical parameters of the animals. In addition, it is very important to check the absolute amino acid values, because of the possible misprint in reference [28]: the amino acid content in milk must be in the range of the “g/kg” instead of the “mg/kg” [28]. A group led by the author of [2,50,51] obtained AOA values for each specific amino acid using the amperometric method as an equivalent to the standard (gallic acid [51]). Comparing the AOA values for each individual amino acid, we obtain the maximum value for tryptophan (35.9 a.u.), which is almost three times and nine times higher than that for tyrosine (13.2 a.u.) and phenylalanine (3.9 a.u.), respectively. Interestingly, in our study, similar AOA values for cysteine (2.00 a.u.) and methionine (2.57 a.u.) are noticeably lower than for aromatic amino acids. It is known that amino acids can generate electrochemical signals, particularly through oxidation, via their electroactive side chains. Amino acids such as tyrosine, tryptophan, cysteine, histidine, and methionine make the most significant contributions to the electrochemical signal. For example, tyrosine and tryptophan exhibit distinct oxidation peaks near +0.78 V and +0.76 V, respectively [217,218]. The dependence of the electrochemical behavior of amino acids on the pH of the medium, the type of electrode surface, and the measurement conditions plays a crucial role [219].

This is not surprising; if we analyze the mechanisms of their oxidation at the electrode, then aromatic amino acids actually have the highest electrochemical potential of amino acids in mediating reactions.

### 4.4. Carbohydrates

Carbohydrates are a large class of natural biologically active substances with the general formula (CHOH)*_n_*, where *n* = 3–7 for the most important monosaccharides. In milk and dairy products, the main carbohydrate component is lactose, a disaccharide consisting of glucose and galactose residues, while free glucose and galactose are contained in cow’s milk at a level of only 50 and 20 mg/kg, respectively. Lactose is contained in cow’s milk at a level of 47–48 g/kg [28]; up to 5% [29]; or in the range of 4.4–5.2 g/100 g [32]. It is of great importance in the nutrition of young animals and children. The functions of carbohydrates are diverse, as they (a) represent the main energy material for the vital activity of the organism (energy function); (b) are part of glycoproteins, nucleic acids, various cellular components, etc. (structural function); (c) are important reserve biologically active substances of the body, as they can be deposited in the form of glycogen in most organs and used when necessary, turning into fats and other biologically active substances (storage or reserve function); d) are part of antibodies (blood γ-globulins), which exhibit immunological properties (protective function); and e) are capable of exhibiting special biological activity as blood anticoagulants (heparin), cardiac glycosides (therapeutic drugs), and “sorbents” that bind water and ions (mucopolysaccharides) [2,28,29,30,31,32]. However, there is no reliable data on the contribution of carbohydrates to milk’s antioxidant system.

Typically, authors conduct research not on the antioxidant activity of lactose itself but on the effectiveness of adding antioxidants (as supplements to feed) on lactose level [18,220]. For example, the study [220] examined the effectiveness of both separate (experimental groups 1 and 2) and combined feeding of two antioxidants (experimental group 3). This resulted in the highest protein and lactose content in the milk of cows in group 3, which significantly (*p* > 0.95) outperformed control cows by up to 7.5% in terms of lactose levels and other milk indices [220].

The only exception occurs when a novel lactose derivative (NLD-6) is synthesized, as exemplified by introducing two amino groups in the places of two hydroxyl groups at the C-6 of lactose [221]. The NLD-6 shows better scavenging activity (in vitro) against hydrogen peroxide (IC_50_  <  0.1 mg·mL^−1^), hydroxyl radicals (IC_50_ 0.55 mg·mL^−1^), and DPPH radicals (IC_50_ 0.37 mg·mL^−1^) than initial lactose [221]. Thus, the introduction of amino group through chemical modification significantly enhances the free antioxidant activity of lactose. Moreover, the authors [222] presented the antioxidant potential of acetylated milk oligosaccharides (A-MOs) and deacetylated oligosaccharides (D-MOs), sourced from “Rathi cow” milk, for neonatal nutrition and human health. Such “derivatization strategy” can be considered as an effective tool with which to broaden utilization of lactose and oligosaccharides, as well as some other BASs, but we will not pursue this topic further.

### 4.5. Lipids

**Figure 4:**
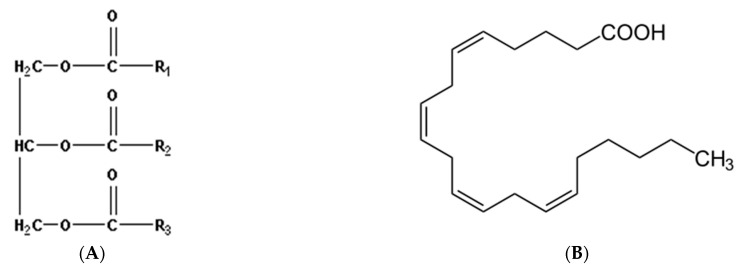
Molecules of some lipids: (**A**) general formula of lipids, where R1, R2, R3 are higher monobasic fatty acids; (**B**) arachidonic acid.

Lipids (or “fats”, especially describing milk “triacylglycerols” in total) are a group of organic substances (of both synthetic and biological origin), the main properties of which are the ability to dissolve completely in organic solvents (chloroform, hexane, benzene, ether, alcohols, etc.) but not in water [22,23,24,28,29,30,42,43,44,50,51,52,53,54]. The exceptionally poor solubility of lipid molecules in polar solvents is associated with an insufficient content of atoms with a polarizing electron shell (O, N, P and others) in comparison with the predominant number of C and H atoms [28,29,30,50,51,52,53,54]. Molecules of simple lipids (Figure 4) consist of residues of monobasic carboxylic acids (MBCA) or, in particular, fatty acids (FAs), including the very important arachidonic acid (Figure 4B) and alcohol (for example, a “trihydroxyl alcohol–glycerol”), linked together by ester bonds (Figure 4A) [30].

Carboxylic acids that have two or more double bonds (-CH=CH-) separated by a single methylene bridge (–CH_2_–) are called “methylene-shared polyenes” [28,29,30,50,51,52,53,54]. This moiety is sometimes referred to “as divinylmethane” [28,29,30,50,51,52,53,54]. Such bonds represent an optimal balance between activity and stability and are therefore presented in major PUFAs such as linoleic, linolenic, and arachidonic acids (former called “vitamin F”) [28,29,30,50,51,52,53,54]. This is important to understand correctly, as the incorrect term “conjugated bonds” is often used in the literature [223] instead of the term “methylene-shared bonds” [30]. In the latter case, these are bonds that form a single delocalized electron cloud in the molecule as a result of the overlap of unhybridized p-orbitals. Conjugation leads to a sharp increase in the oxidation susceptibility of such carboxylic acids due to the redistribution of electron density in the molecule and the alignment of bond lengths and energies. Examples of conjugated double bonds in lipid biochemistry are rare, but they have an important place in biotechnology [42,43,44,50,51,52,53,54]. The simplest form can be considered as sorbic acid (trans,trans-2,4-hexadienoic acid), which contains a conjugated double bond in the molecule (CH_3_—CH=CH—CH=CH—COOH) and is widely used as a food preservative (approved food additive E200 in Russia). Formally, sorbic acid is not considered an antioxidant, but the author personally believes that it exhibits particular antioxidant properties through an alternative pathway, as it oxidizes faster and more easily than other unsaturated fatty acids, which are more important in mammal metabolism. One confirmation of the author’s assumption is its frequent use to prevent food spoilage (it inhibits the activity of mold, yeast, and even some bacteria). For example, sorbic acid is officially used in the production of condensed milk to prevent mold, as well as in cheeses, sour cream, cottage cheese, and in the production of packaging materials for dairy products [30,42,43,44,50].

These unique properties determine the important roles of lipids in animal cell processes and in special secretions, such as milk. In animals, up to 50 types of fatty acids are used by the mammary gland for fat synthesis or enter the milk directly from the blood. Fresh milk contains a very low concentration of free fatty acids, and when their concentration increases, the milk acquires a rancid odor. The total fat in milk can be in the range of 3–4% depending on the cow breed, lactation period, seasonal variations, etc. [2,9,28,29,30,50,51,52,53,54]. It is important to check the fatty acid content in milk lipids. Usually, saturated fatty acids (or SFAs, with no double bonds and 660–690 g/(kg milk fat) [223]) such as myristic, palmitic, and stearic acids make up two thirds of milk fatty acids. Palmitoleic and oleic acids are the most abundant among the monounsaturated fatty acids (MUFAs, with one double bond and 275–305 g/(kg milk fat) [223]). There are a few major polyunsaturated fatty acids (PUFAs, with 2–4 double bonds and 59–82 g/(kg milk fat) [223]). In the interesting paper [223], the authors compare the fatty acid and fat-soluble antioxidant composition of milk from three UK production systems: certified-organic ‘low-input’ (O-LI), non-organic certified ‘low-input’ (NO-LI) and standard ‘high-input’ (HI) organic systems. Milk from both the low-input organic and non-organic systems had significantly higher concentrations of nutritionally desirable FAs and antioxidants—those being conjugated linoleic (60% and 99%, respectively) and α-linolenic (39% and 31%, respectively) acids, α-tocopherol (33% and 50%, respectively), and carotenoids (33% and 80%, respectively)—compared with milk from the high-input system [223]. Milk composition differed significantly between the two low-input systems during the second half of the grazing period only, with milk from non-organic cows being higher in antioxidants and “conjugated” linoleic acid and that from organic cows being higher in α-linolenic acid [223].

Studies of milk powders have shown that phospholipids identified by TLC indicate the presence of different phospholipids. In study [224], the authors documented a correlation between the total yield, H_2_O_2_ scavenging activity, and ferric reducing power; they confirmed its antioxidant nature was not in the order seen earlier in TLC. In conclusion, milk phospholipids present in milk powders play an important key role in both nutritional and technological fields. By measuring “radical scavenging activity”, the least activity was found in the “dairy whitener”, as the percentage of H_2_O_2_ reduced was increased, and the highest activity was found in “glucipro milk powder” [224].

In the pioneering study [225], a detailed investigation was carried out on the antioxidant capacity of raw and thermally processed cow and buffalo milk. The antioxidant activity of milk samples in a linoleic acid system (AOAinLA) was determined by the method prescribed by Osawa and Namiki [226]. The final absorbance was measured at 500 nm on a double-beam spectrophotometer, and BHT was used as a positive control [227]. The results of the free radical scavenging activity in linoleic acid of raw, pasteurized, and boiled milk after 0, 3, and 6 days of storage were as follows. The AALA of raw, pasteurized, and boiled cow milk was 11.7%, 11.3%, and 11.1%, respectively. The AALA of raw, pasteurized, and boiled buffalo milk was 17.4%, 17.1%, and 16.8%, respectively [225]. The AALA of raw, pasteurized, and boiled milk samples after up to 3 days of storage did not differ from fresh samples (0 day). The AALA of samples stored for 6 days in refrigeration was considerably different from fresh samples in raw, pasteurized, and boiled milk. The AALA of dairy products has been reported in the existing literature [225].

Among the biological functions of lipids, one of the most important is their ability to release large amounts of energy (during controlled oxidation), which is used to generate heat and ATP (energy function). However, when lipids undergo undesirable oxidation, reactive radicals can spontaneously form, which is what antioxidants are designed to prevent. In my opinion, neither fatty acids nor even lipids, as a whole, function as classical antioxidants; rather, the function of other antioxidants is to protect fatty acids and lipids from oxidation [2,28,29,30,31,32,132]. This is why the function of lipids is more effective in complex with classical antioxidants (vitamins, enzymes, peptides, etc.).

### 4.6. Final Remarks on Part 4

In the author’s opinion, proteins and peptides make the most significant contribution to the antioxidant system of milk, since the content of the latter in milk is significantly higher than that of classical antioxidants, whereas saccharides or lipids have almost no AOA. In contrast, a “derivatization strategy” can be considered an effective tool with which to broaden the utilization of lactose and oligosaccharides, as well as some other BASs. In the author’s opinion, neither fatty acids nor even lipids, as a whole, function as classical antioxidants; rather, the function of other antioxidants is to protect fatty acids and lipids from oxidation. Moreover, the function of any antioxidant is more effective in complex with some other antioxidants (vitamins, enzymes, peptides, etc.).

## 5. Methods for Studying the Antioxidant Activity of Various Biological Objects

“The oxidant/antioxidant balance” of the body is determined by numerous “oxidation–reduction reactions” (“Ox–Red”) based on the following mechanisms: I) single electron transfer (SET) and II) hydrogen atom transfer (HAT) [2,7,8,9,14,15,16,17,18,19,228,229,230]. Therefore, the general principles for determining any antioxidant’s activity in biosamples [228,229] are based on “Ox–Red” and can be implemented by various biochemical–physical–chemical methods [7,8,9,17,18,19,20,21,50,51,52]. The numerous methods are used for the determination of the total antioxidant activity or AOA of any milk component [231,232,233,234,235,236,237,238,239,240,241,242,243,244,245,246,247,248,249,250,251,252,253] include “Ox–Red” titration; ferric-reducing antioxidant power (FRAP); cupric-reducing antioxidant capacity (CRAC and CUPRAC); 2,2-azino-bis(3-ethylbenzothiazoline-6-sulfonic acid) (ABTS); 1,1-diphenyl-2-picrylhydrazyl hydrate (DPPH); oxygen radical absorbance capacity (ORAC); hydroxyl radical antioxidant capacity (HORAC); total radical-trapping antioxidant parameter (TRAP); and total oxyradical scavenging capacity (TOSC). The classification of methods for measuring milk AOA differs significantly in various publications (for example, [2,7,8,9,19,30,228,235]); therefore, only a limited number of key methods are summarized below (Table 3).

### 5.1. “Ox–Red” Titration

“Ox–Red” titration [229,230,231] is one of the basic chemical methods for determining antioxidant activity [7,8,9,10,13,14,15,16,17,18,19,228]; it uses potassium permanganate as an oxidizing agent (calibrated using the standard, quercetin [230]). The activity indicator (A*, which is proportional to AOA) is measured as the volume of the sample (reducing BASs) in milliliters spent on titration of 1 mL of 0.05 N potassium permanganate solution. The smaller the volume of the sample spent on titration, the higher the AOA of the sample (A* value). Recalculation of the A* (AOA) indicator to the standard—quercetin (in mg/g [230])—is carried out using the corresponding formulas [228,229,230]. The disadvantages of this method are the need for fairly large volumes of the sample solution in order to improve the accuracy of titration and the “visual fixation of the equivalence point”, which increases the error within the analysis. It is significant that an identical method of photometric titration “with optical detection” has already been developed [231], overcoming the disadvantage of “visual fixation of the equivalence point” [228,229,230].

### 5.2. Spectroscopic Methods

#### 5.2.1. “FRAP” Method

One of the basic methods for determining the total antioxidant activity of complex objects (including biosamples) is the spectroscopic method “FRAP”. The “FRAP” method is based on the ability of antioxidants to reduce the “iron(III) ion” [232,233,234,235]. In the photometric determination of the ability of antioxidants to reduce Fe(III), various indicator systems are used, which include a colored complex with the reduced form of iron (Fe(II) [232].

Antioxidant + Fe(III) ↔ Fe(II) + Oxidized antioxidant (7)

Examples of such compounds are “tripyridyltriazine, potassium hexacyanoferrate [232], 2,2′-dipyridyl, o-phenanthroline” [233]. Indicator systems with the last two reagents are characterized by higher values of “Red–Ox potentials” (0.97 V and 1.19 V, respectively) [232,233,234], which makes it possible to expand the range of biologically active substances that can be determined. The choice of wavelength at which the AOA determination is carried out depends on the specific indicator system. A number of spectrophotometric methods for determining the AOA of food products based on the Fe(III)/Fe(II) oxidation–reduction reaction have been described previously [232,233,234,235]. For example, the well-known publications [232,233,234,235,236] are describing methods for determining antioxidants (e.g., ascorbic acid using the color intensity of Fe(II)—pyridyl-2,6-dicarboxylic acid [232] and Fe(II)—ferrocene [233] (at λ = 517–519 nm) or Fe(II)—o-phenanthroline (1,10-phenanthroline) [234] (at λ = 490 nm) systems). Based on the study of the main individual antioxidants (using the Fe(III)/Fe(II)-o-phenanthroline indicator system), they were grouped in the following descending order of AOA: quercetin > gallic acid > rutin ≥ protocatechuic acid (pyrocatechuic acid) = catechol = ascorbic acid [234]. The choice of ascorbic acid as a standard substance for determining the antioxidant activity (AOA) of food products is substantiated by the authors of [234]. The «FRAP» method for assessing the AOA of food products has fairly good sensitivity and is “metrologically substantiated” [50]. However, this method cannot determine «sulfhydryl (or SH)-containing antioxidants», such as glutathione and cysteine [232,233,234,235]. Therefore, the valuable information obtained by this method has some limitations.

#### 5.2.2. “CUPRAC” and “CRAC” Methods

In addition to the reduction of Fe(III), the methods based on the reduction of the Cu(II) («CUPRAC» (cupric ion-reducing antioxidant capacity at λ = 450 nm) [235,237]) and Ce(IV) by antioxidants («CRAC» (ceric-reducing antioxidant capacity at λ = 320 nm)) [236] can be used. It should be noted that insufficient study of the FRAP and CRAC complexes for milk AOA and the laboriousness of obtaining calibrations prevent the widespread use of these methods.

Herein, it is worth presenting only a brief analysis of the CUPRAC modifications, since an extensive analysis has been carried out in significant works by Munteanu et al. [235], Ferreira et al. [236], and Özyürek et al. [237]. Certain advantages of CUPRAC over other methods are as follows: (A) the electronic structure of Cu(II) ensures fast kinetics of oxidation of thiol-type antioxidants (while other methods such as FRAP do not allow this); (B) the redox reaction leading to the formation of colored forms is carried out in a buffer with pH 7.0 (which is very important for biospecimens), in contrast to the acidic conditions (pH 3.6) of FRAP or the basic conditions (pH 10.0) of the total phenolic content (TPC); (C) CUPRAC reagent is highly selective due to its lower oxidation–reduction potential than an iron(III)–iron(II) pair (in the presence of phenanthroline or similar ligands); (D) the reagent is more stable and readily available than other chromogenic reagents (e.g., ABTS, DPPH); (E) this method is versatile in that it allows measurement of both hydrophilic and lipophilic antioxidants (e.g., β-carotene and α-tocopherol); (F) the reliability of the method is confirmed by the fact that the composition of the air environment, humidity, and sunlight do not affect the reaction of CUPRAC with antioxidants (in contrast to free radical-type reagents such as DPPH); (J) this method has high sensitivity and linear response over a wide range (in contrast to other methods giving polynomial curves)—its molar absorptivity is (7.5–9.5 × 10^3^ *n*) L·mol^−1^·cm^−1^, which is high enough for the sensitive determination of biologically important antioxidants; (G) it is positive that the total absorbance values of antioxidants determined by CUPRAC are perfectly additive (i.e., the total absorbance of a mixture is equal to the sum of the total absorbance values of its components), whereas in various other similar methods (e.g., FRAP reactions), thiol compounds have shown no additivity with a number of polyphenolic compounds; and (H) this method is easily and widely applicable in routine laboratories using standard colorimeters, without requiring sophisticated equipment and skilled operators. It is also applicable in biological, food, and medical industries [235,236,237].

#### 5.2.3. “DPPH” and “ABTS” Methods

A recent review by Gulcin and Alwasel [238] provides a critical description of some of the most important methods (including “DPPH” and “ABTS”) used to determine antioxidant activity, their mechanisms of detection, applicability, advantages, and disadvantages. This review analyzed numerous studies, concerning adaptations of the DPPH radical scavenging method to various assays, determination of antioxidant activity indices, standardization conditions, and optimization of measurement methods. In addition to key sections on definitions, chemicals, and the basic principles of scavenging residual DPPH radicals, the methods and technical details of the method are described. Furthermore, this method project outlines and discusses key areas of application of the DPPH method in the food and pharmaceutical industries. However, despite rapid advances in instrumentation and analysis, this method does not prevent harmful changes [238]. In practical terms, for a detailed comparative examination of the DPPH and ABTS methods, one of the few existing studies is that published by Akhtar et al. [239], where commercial dry whey for food purposes “subjected to electroactivation” (EADW) was investigated. After 10–20–30–40 min of EADW treatment at 800 mA, the DPPH radical absorbance capacity (RAC) increased gradually in EADW samples, reaching approximately 37–40-56–55% (control curve), whereas after 50–60 min of EADW treatment, the DPPH RAC decreased sharply in the same EADW samples, reaching appr. 45–32% (control curve [239] Moreover, under the same conditions (after 10–20–30–40 min), the DPPH RAC increased gradually in EADW samples (with an “aging time of 48 h” or long-term incubation), reaching approximately 47–55–86–71% (experimental curve II), whereas after 50–60 min of EADW treatment, the DPPH RAC decreased sharply in the same EADW samples, reaching approximately 38–31% (experimental curve II) [239]. There are “some intermediate data” (experimental curve I) for the same EADW samples but with an “aging time of 24 h” [239]. So, in any case, there is a parabolic tendency in the DPPH RAC values for all experimental and control curves. In contrast, there is a common exponential tendency (with some pronounced fluctuations) for the ABTS values over 10–60 min of EADW treatment. This ABTS tendency is even more pronounced for experimental curve I (which reaches a maximum TEAC value of ~0.24 mg/L) compared to the control curve (with a maximum TEAC value of ~0.20 mg/L) or experimental curve II (which has a maximum TEAC value of ~0.17 mg/L), where TEAC means “trolox equivalent antioxidant activity” [239].

It is positive that the ABTS method requires minimal processing, is extremely versatile at various pH values, allows for the selection of different wavelengths to prevent spectrophotometric interference, can be used for both hydrophilic and lipophilic measurements, and is easily adapted to high-throughput methods (microplate, HPLC, etc.), thereby satisfying virtually all requirements for optimal AOA analysis [240]. The common disadvantages of the DPPH and ABTS methods include the lack of natural physiological radicals, large sample size, and relatively slow antioxidant response [240]. However, both these methods have their essential advantages and remain more useful in modern analyses compared to the other methods mentioned above.

### 5.3. Coulometric Method

A number of studies assess the antioxidant properties of milk samples from various animals using “coulometry” [241,242,243,244]. This method is based on coulometric titration of their solutions with “electrogenerated titrants” (such as Cl_2_, I_2_, Br_2_), the generation of which is carried out from solutions of their salts [241,242,243,244]. The “end point of titration” is recorded in an “amperometric system with two platinum electrodes” [242,243]. The antioxidant capacity is calculated “as the amount of electricity spent on titration (per 100 g of biologically active substance)” using the corresponding formulas [242], which take into account the current strength (I, Amperes); the time to reach the end point of titration (t, sec.); and the volumes of antioxidant and aliquot (i.e., V_1_ and V_2_, ml, respectively) [242]. It is interesting that we have an exact description of the analysis method: “the titrant is electrogenerated from a 0.1 M solution of potassium iodide in a phosphate buffer solution (pH = 9.8) on a platinum electrode at a constant current of 5.0 mA” [242]. This method is distinguished by the fact that it uses the interaction of the studied BASs with a “coulometric titrant—hypoiodite ions formed during the disproportionation of electrogenerated iodine in an alkaline medium”. It is positive that a modification of this method for determining AOA “in plant materials in terms of tannin by coulometric titration” has already been patented [242], and its application for analysis of milk and other biological liquids has been described [241,242,243,244].

### 5.4. Voltammetric and Potentiometric Methods

Since the determination of the “antioxidant/oxidant balance of the body” is based on electrochemical principals, voltammetric and potentiometric methods of assessing the specified AOA parameters can be considered “most fully corresponding to the nature of the phenomenon” [245,246,247,248,249]. Electrochemical methods are characterized by high sensitivity and “fast response”. For example, a number of authors believe that “the detection limit of polyphenols and flavonoids is at the level of 10^−9^–10^−12^ g” [7,8,9,19,26,245,246,247,248,249], which is a very good result.

The well-known method of “cyclic voltammetry” (CVA) and its modifications are actively used to determine the antioxidant activity of various solutions of pure and mixed BASs, “plant extracts”, etc. [245,246,247,248,249,250]. In this case, the antioxidant properties of biological samples are assessed as a combination of several CVA parameters. For example, the “parameter E_1/2_” or “half-peak potential” of the first and second wave peaks (at about 400 and 900 mV, respectively [246]) is necessary to identify correlations with a particular substance “forming current peaks” in the blood serum samples. It is reported [246] that the first wave peak can be attributed to the presence of AA and uric acid, whereas the second wave peak can presumably be attributed to GSH or similar peptide antioxidants [246].

The results of studies [245,246,247,248] have shown that “the number of anodic waves and the value of E_1/2_” are significantly affected not only by the features of the CVA device (i.e., the material and shape of the working electrode, etc.) but also by the composition of the solution or “bioextract” [245,246,247,248]. For example, the parameter E_1/2_ was successfully used to classify AOA into the three types of model solutions: amino acids (CYS, HIS, TRP, TYR), phenolics (catechin or rutine), and mixed phenol–AA solutions [249,250]. The main disadvantage of this method is that “the position of the current maxima on the anodic curves” obtained for various bioassays and even animal biosamples may shift back and forth “within more than 200 mV” [245,246,247,248,249,250]. In the case of such a shift or “extra anodic waves”, additional analyses (such as HPLC [248]) are required to confirm the qualitative and quantitative composition of antioxidants. An interesting modification of the CVA method (using the “mercury film electrode”) for determining the activity of antioxidants using the first wave peaks is being developed [248]. However, all these and the majority of other works [245,246,247,248,249,250] present the results of CVA in model systems, blood serum, plant extracts, etc. [245,246,247,248,249,250]. The detection of only dairy fouling, but not milk antioxidants, by CVA and square wave voltammetry (SWV) using platinum-based interdigitated microelectrodes is reported in another interesting work [251]. The authors [252] concluded that the electrochemical method (CVA) is faster than HPLC (which included a pretreatment step), providing an inexpensive and simple method for the reliable analysis of uric acid in milk (based on the main anodic peak at 330 mV [252]). Quite simple and reasonably informative is the “potentiometric method with a mediator system” [253,254,255,256], which is adapted to the analysis of a wide range of similar bioassays. However, no systematic quantitative determination of the major milk antioxidants using cyclic voltammetry or potentiometric methods has been reported in the available literature.

### 5.5. Amperometric Method for Studying the Total Antioxidant Activity of Milk

Among all the antioxidant indicators of milk, the simplest and most informative is the determination of its total amount of water-soluble antioxidants (TAWSA) using the amperometric method, which is confirmed by numerous publications and data [2,10,11,12,19,27,28,29,30,50,51,52,53,54,257,258,259]. Many other methods of analysis are “indirect”, since they measure the “inhibition of reaction mixtures containing free radicals” formed as a result of certain processes and reactions (mentioned above in Section 5.1, Section 5.2, Section 5.3 and Section 5.4). Amperometric detection consists of measuring the electric current in the cell that occurs during the “Ox–Red” reactions of the analyzed substance on the surface of the working electrode when a potential of +1.3 V is applied to it. The sensitivity of the amperometric detector is very high due to its low noise levels (about 10^−12^ Ampers). The working electrode material is glassy carbon, which is the most versatile in determining polyphenolic and other antioxidant compounds [50,51,52,53,54]. As a standard for measuring the TAWSA of the samples, we used “working solutions” of gallic acid (100 mg/dm^3^) with a mass concentration from 0.2 to 4.0 mg/mL, as described in detail in [10,11,12,50,51,52,53,54]. A solution of orthophosphoric acid 2.2 mM/mL was used as an eluent. The signal from the analyzed milk samples was displayed as peaks on the calibration graph; the peak area (nA/s) was used for further recalculation. In the case of analyzing biological fluids, including milk, the signal (X_i_) exceeded the signal of the “calibration” solution (4.0 mg/dm^3^ of gallic acid), so the milk samples were preliminarily diluted 20 times (for example, 0.1 mL of the sample and 1.9 mL of bi-distilled water) [10,11,12]. The mass concentration of antioxidants (X, mg/g) is calculated as an equivalent to gallic acid using the calibration graph, taking into account possible dilution [10,11,12,50,51,52,53,54].

For example, in works [10,11,12,257,258,259], the following average values of TAWSA in milk samples were obtained depending on the lactation period (for black-and-white and Holstein breeds of cows): 14.83 mg/L (151–165 days); 14.27 mg/L (166–180 days); 12.50 mg/L (181–210 days); 16.78 mg/L (211–225 days); and 13.48 mg/L (226–240 days) [11,257]. Averaging these data, we obtained an average value of TAWSA of 14.37 mg/L in cows’ milk samples (with min. values of 12.50 mg/L and max. values of 16.78 mg/L) [11,257]. In the recent papers [258,259], statistically reliable data on TAWSA values (13.41 mg/L and 21.29 mg/L) were obtained for the first time in groups with low (16.6 L) and high (30.2 L) daily milk yield (groups 1 and 2, respectively) [258]. It is important that for high-yielding cows, the range of min. and max. TAWSA values was from 12.53 mg/L to 28.31 mg/L, i.e., the upper limit of values for such cows should be raised to about 30 mg/L. The authors determined high correlation coefficients (CCs) for TAWSA with the major milk parameters. There was a positive CC with the values of fat and protein in milk, but a negative CC with the total milk yield of cows [259]. In the latter case (negative CC), this indicates the inability of the organism to synthesize fat and protein molecules in the volumes that yield high TAWSA values and are necessary to maintain milk TAWSA in cows with high total milk yields. For a more concise and visual presentation of the results, it was rational to evaluate the ratio of TAWSA to milk yield using the following coefficients: 0.8 and 0.7 for groups 1 and 2, respectively. The ratio of TAWSA to fat (protein) content was 2.9 (3.2) and 5.8 (6.4) for groups 1 and 2, respectively [259].

Thus, the author proposes applying an indicator such as TAWSA (with values from 10 to 30 mg/L for “raw milk” obtained for the black-and-white and Holstein breeds of cows) as a recommended (“reference”) parameter in animal husbandry.

## 6. Conclusions

There is growing interest in antioxidants, particularly for improving the quality of agricultural products, extending food’s shelf life, and preventing the known harmful effects of free radicals on the human metabolism. In all cases, antioxidants of plant or animal origin (including milk antioxidants) are far preferable to synthetic analogues. There are a fairly large and varied number of antioxidants in milk (as summarized in Table 1 and Figure 2); these are vitamins, proteins (including enzymes), peptides and amino acids, fatty acid residues of lipids, etc., and they fight with reactive oxygen species. This is why there is currently explosive growth in the development and use of assays for assessing the effectiveness of antioxidants on the human metabolism and food systems. Numerous biochemical and biophysical methods for measuring antioxidant effects currently exist. This article provides a critical overview of the most important bioanalytical assays used to determine antioxidant activity and their mechanisms and applicability, advantages, and disadvantages (primarily for antioxidants of milk and dairy products).

To date, current technical specifications for milk and dairy products in Russia and many other countries lack either individual indicators or a single overall indicator for their antioxidant activity. Among all the antioxidant indicators of milk, the simplest and most informative is the detection of the total amount of water-soluble antioxidants (TAWSA), which is confirmed by comparison of numerous publications and practical results of various methods (as summarized in this review). It is important to emphasize that the TAWSA of milk is an “integral characteristic” of the most valuable biologically active substances in milk that possess antioxidant properties. It is not associated with the content of a specific compound but reflects the properties of the entire milk antioxidant system, and it therefore characterizes the most important interrelations in cow metabolism.

Thus, today, the range of methods for determining the total antioxidant activity of milk is very diverse, but the lack of unified criteria and methodology in this area makes it difficult to compare the results of different studies. Therefore, it is advisable to use the TAWSA method to assess the antioxidant activity of milk as an “integrated indicator.”

## Figures and Tables

**Table 1:** A list of the key biologically active substances in animal milk and their antioxidant properties.

№	BAS *, Groups, Classes	Specific Compound	Molecular Mass (MM), Type of Reaction	Parameters and Conditions
1	Enzymes, class oxidoreductases	Xanthine oxido- reductase, EC 1.17.3.2.	MM ~270 kDa; catalyzes the oxidation of hypoxanthine to xanthine and then to uric acid	Protein is a dimeric complex “molybdenum-flavo-enzyme”, a component of the membranes of fat globules
2	Enzymes, class oxidoreductases	Lactoperoxidase as NADH-peroxidase, EC 1.11.1.X	It is believed that the antimicrobial ability of lactoperoxidase is synergistic with lactoferrin and lysozyme	Iron-containing glycoprotein; the catalytic center contains protoporphyrin IX, covalently linked (S-S-bridge) to the polypeptide chain. Other examples: bromide (Br) → hypobromite (BrO) iodide (I) → hypoiodite (IO)
3	Enzymes, class oxidoreductases	Superoxide dismutase (SOD), EC 1.15.1.1	MM ~32.5 kDa; catalyzes the dismutation of superoxide (superoxide radical) into oxygen and hydrogen peroxide. SOD has a high catalytic reaction rate (~10^9^ M^−1^ s^−1^).	The superoxide dismutation reaction under the action of SOD: (1) M^(*n* + 1)+^ − COД + O_2_^−^ → M^n+^ − COД + O_2_ (2) M*^n^*^+^ − COД + O_2_^−^ + 2H^+^ → M^(*n* + 1)+^ − COД + H_2_O_2_., where M can be (Cu (*n* = 1); Mn (*n* = 2); Fe (*n* = 2); Ni (*n* = 2).
4	Enzymes, class oxidoreductases	Catalyse, EC 1.11.1.6	MM ~250 kDa; catalyzes oxidation-reduction reaction: 2H_2_O_2_→2H_2_O + O_2_.	In a reaction, two molecules of hydrogen peroxide form water and oxygen
5	Peptides, tripeptides	Glutathione or γ-glutamylcysteinylglycine	MM~307 Da, C_10_H_17_N_3_O_6_S, tripeptide or (2-amino-5-{[2-[(carboxymethyl)amino]-1-(mercaptomethyl)-2-oxoethyl]amino}-5-oxopentanoic acid	Glutathione in its reduced form can function as an antioxidant in several ways: by chemically interacting with singlet oxygen, superoxide, and hydroxyl radicals or by directly destroying free radicals, and by stabilizing membrane structure
6	Amino acids, SH-containing compounds	Cysteine, methionine	CYS *, MM 121.16 Da, C_3_H_7_NO_2_S, MET *, MM 149.21 Da C_5_H_11_NO_2_S	Cysteine and methionine are among the most powerful antioxidants; their antioxidant effect is enhanced in the presence of vitamin C and selenium
7	Lipids, monounsaturated fatty acids (MUFAs)	Oleic acid, palmitoleic acid	MM 282,46 Da C_18_H_34_O_2_ C_17_H_33_COOH MM 256.5 Da C_16_H_32_O_2_ C_15_H_31_COOH	MUFAs react with bases, oxidizing agents, reducing agents, and also with oxygen to form lipid peroxidation products (LPO)
8	Lipids, polyunsaturated fatty acids (PUFAs)	Linoleic acid linolenic acid arachidonic acid	MM 280.45 Da C_18_H_32_O_2_ MM 278,43 Da C_18_H_30_O_2_ MM 304.47 Da C_20_H_32_O_2_	PUFA react with bases, oxidizing agents, reducing agents, and also with oxygen to form lipid peroxidation products (LPO)
9	Vitamins, fat-soluble vitamins	Vitamins A, E, K	α-tocopherol MM 430.7 Da; β-tocopherol MM 416.7 Da; γ-tocopherol MM 416.7 Da; δ-tocopherol MM 402.7 Da	Some of the most powerful antioxidants; they are light yellow oils soluble in acetone, EtOH, CHCl_3_, and diethyl ether and insoluble in H_2_O
10	Vitamins, water-soluble vitamins	Vitamin C, vitamin B_6_, vitamin PP	MM 176.12 Da C_6_H_8_O_6_. MM 169.18 Da C_8_H_11_NO_3_ MM 123.11 Da C_6_H_5_NO_2_	Some of the most powerful antioxidants
11	Low-molecular-weight antioxidants, phenols, poly-phenols	Tocopherol acetate, eugenol, pyrocatechin, gallic acid	MM 472.8 (430.7) Da C_31_H_52_O_3_. MM 164.20 Da C_10_H_12_O_2_ MM 110.11 Da C_6_H_6_O_2_ MM 170.12 Da C_7_H_6_O_5_	α-tocopherol acetate (light yellow oil), λ_max_ 292 nm, ε 3260, eugenol, pyrocatechol, and gallic acid are bioactive compounds; they are derivatives of phenolic acid and have strong antioxidant properties

Notes *: BASs—biologically active substances; MET—methionine; CYS—cysteine.

**Table 2:** Amino acid content in milk proteins *.

Amino Acid	TP_1_ g/100 g	TP_2_ mg/100 g	TP_3_ g/100 g	TP_4_ mg/kg	AOA a.u.
ASP	0.23–0.26	219	0.232	0.7–2.9	-
THR	0.11–0.12	153	0.145	0.8–1.4	-
SER	0.17–0.19	186	0.175	0.8–2.8	-
GLU	0.59–0.67	509	0.651	4.0–32.0	-
GLY	0.04–0.05	47	---	2.0–15.0	-
ALA	0.12–0.14	98	0.100	1.4–2.9	-
VAL	0.20–0.23	191	0.199	0.6–1.5	-
ILE	0.16–0.19	189	0.180	0.3–0.9	-
LEU	0.30–0.35	283	0.326	0.2–0.9	-
TYR	0.14–0.17	184	0.154	0.1–0.5	++
PHE	0.15–0.18	175	0.167	0.1–0.3	+
HIS	0.08–0.10	90	0.097	0.7–5.5	-
LYS	0.26–0.30	261	0.273	2.8–8.1	-
ARG	0.12–0.14	122	0.121	1.4–4.5	-
PRO	0.21–0.25	278	0.327	2.5–5.4	-
TRP	---	50	0.048	---	+++
MET	0.12–0.14	83	0.113	0.3–0.7	+
CYS	0.03–0.04	27	0.028	0.1–5.8	+

Note *: TP_1_—personal data of the author’s group [2], TP_2_—average data [32], TP_3_.—average data [20], TP_4_—interval of data [28], ASP—aspartic acid, THR—threonine, SER—serine, GLU—glutamic acid, GLY—glycine, ALA—alanine, VAL—valine, ILE—isoleucine, LEU—leucine, TYR—tyrosine, PHE—phenylalanine, HIS—histidine, LYS—lysine, ARG—arginine, PRO—proline, TRP—Tryptophan, MET—methionine, CYS—cysteine; AOA values for amino acid: - no activity, + low activity, ++ medium activity, +++ high activity.

**Table 3:** Selected key methods for studying antioxidant activity.

№	Method and Reaction Type	Substances or Indicators	Medium and Reagent	Conditions
1	“Ox–Red” titration	Major WSA *, including quercetin (in mg/g)	0.05 N potassium permanganate solution (in ml), aqueous medium	1. “Visual fixation of the equivalence point” [229,230] 2. “Photometric titration according to Levetal with optical detection” [231]
2	«FRAP» spectroscopy	Ascorbic acid, glutathione and cysteine	Fe(III)/Fe(II)	Assessment by the intensity of color of the complex Fe(II)-pyridyl-2,6-dicarboxylic acid, Fe(II)-ferrocene [232,233,234,235]
3	«CUPRAC» spectroscopy	Ascorbic acid, glutathione and cysteine	Ce(II) (λ = 450 nm)	Assessment by the intensity of color of the Cu^2+^ complex (λ = 450 nm) [233,235] (2011). A comprehensive review of CUPRAC methodology: Analytical Methods. 3. 2439–2453. 10.1039/C1AY05320E.
4	«CRAC» spectroscopy	Ascorbic acid, glutathione and cysteine	Ce(IV) (λ = 320 nm)	«CRAC» “oxidation–reduction reaction” [233,236]
5	DPPH spectroscopy	Major WSA *	1,1-diphenyl-2-picryl-hydrazyl hydrate (λ = 517–519 nm)	DPPH radical scavenging assay [237,238]
6	ABTS spectroscopy	Major WSA *	2,2-Azino-bis(3-ethylbenzothiazoline-6-sulfonic acid) (λ = 320 nm)	ABTS/TAC methodology [238,239]
7	Coulometry or coulometric titration	Major WSA *	0.1 M potassium iodide solution in phosphate buffer solution (pH = 9.8) on a platinum electrode	Electrogenerated titrants are hypoiodite ions formed by disproportionation of electrogenerated iodine in an alkaline medium [240,241,242,243]
8	Voltammetry or «cyclic voltammetry»	Major WSA *	Process of electro-reduction (ER) of oxygen on a mercury film electrode	Process of electro-reduction (ER) of oxygen on a mercury film electrode [244,245,246,247,248,249]
9	Potentiometric method	Major WSA *	K_3_[Fe(CN)_6_]/K_4_[Fe(CN)_6_] system nMeO_x_L + AO = nMeRedL + AOO_x_	гдe MeO_x_L—“oxidized form of metal and ligand”; MeRedL—reduced form of metal and ligand; AO—antioxidant being determined; AOO_x_—the oxidation product of this antioxidant [250,251,252,253]

Note *: WSA—water-soluble antioxidant.

## Data Availability

Data are available at the Federal Research Center for Animal Husbandry named after Academy Member L.K. Ernst website: https://www.vij.ru/institut/struktura-organizatsii/nauchnye-podrazdeleniya/gruppa-analiticheskoj-biokhimii-2 (accessed on 30 September 2025).
